# Tumour microenvironment‐based molecular profiling reveals ideal candidates for high‐grade serous ovarian cancer immunotherapy

**DOI:** 10.1111/cpr.12979

**Published:** 2021-01-31

**Authors:** Xiaofan Lu, Caoyu Ji, Liyun Jiang, Yue Zhu, Yujie Zhou, Jialin Meng, Jun Gao, Tao Lu, Junmei Ye, Fangrong Yan

**Affiliations:** ^1^ State Key laboratory of Natural Medicines China Pharmaceutical University Nanjing China; ^2^ Research Center of Biostatistics and Computational Pharmacy China Pharmaceutical University Nanjing China; ^3^ Department of Biostatistics The University of Texas MD Anderson Cancer Center Texas USA; ^4^ Division of Gastroenterology and Hepatology Key Laboratory of Gastroenterology and Hepatology Ministry of Health Renji Hospital School of Medicine Shanghai Jiao Tong University Shanghai Institute of Digestive Disease Shanghai China; ^5^ Department of Urology The First Affiliated Hospital of Anhui Medical University Hefei China; ^6^ Institute of Urology & Anhui Province Key Laboratory of Genitourinary Diseases Anhui Medical University Hefei China; ^7^ Department of Urology University of Rochester Medical Center Rochester NY USA

**Keywords:** high‐grade serous ovarian cancer, immune‐specific subtype, immunotherapy response, molecular classification, tumour immune microenvironment

## Abstract

**Objective:**

Due to limited immunological profiles of high‐grade serous ovarian cancer (HGSOC), we aimed to characterize its molecular features to determine whether a specific subset that can respond to immunotherapy exists.

**Materials and Methods:**

A training cohort of 418 HGSOC samples from TCGA was analysed by consensus non‐negative matrix factorization. We correlated the expression patterns with the presence of immune cell infiltrates, immune regulatory molecules and other genomic or epigenetic features. Two independent cohorts containing 482 HGSOCs and in vitro experiments were used for validation.

**Results:**

We identified immune and non‐immune groups where the former was enriched in signatures that reflect immune cells, infiltration and PD‐1 signalling (all, *P* < 0.001), and presented with a lower chromosomal aberrations but increased neoantigens, tumour mutation burden, and microsatellite instability (all, *P* < 0.05); this group was further refined into two microenvironment‐based subtypes characterized by either immunoactivation or carcinoma‐associated fibroblasts (CAFs) and distinct prognosis. CAFs‐immune subtype was enriched for factors that mediate immunosuppression and promote tumour progression, including highly expressed stromal signature, TGF‐β signalling, epithelial‐mesenchymal transition and tumour‐associated M2‐polarized macrophages (all, *P* < 0.001). Robustness of these immune‐specific subtypes was verified in validation cohorts, and in vitro experiments indicated that activated‐immune subtype may benefit from anti‐PD1 antibody therapy (*P* < 0.05).

**Conclusion:**

Our findings revealed two immune subtypes with different responses to immunotherapy and indicated that some HGSOCs may be susceptible to immunotherapies or combination therapies.

## Graphical abstract

A comprehensive profiling of high‐grade serous ovarian cancer based on multifarious molecular features identified an immune group in high‐grade serous ovarian cancer that contains two robust microenvironment‐based subtypes with distinct likelihoods of response to immunotherapies that may also represent ideal immunotherapy candidates.

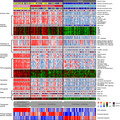

## 1 INTRODUCTION

Ovarian cancer is the fifth leading cause of cancer‐related death among gynaecologic cancers in the United States. In 2019, approximately 22 530 new ovarian cancer cases and 13 980 deaths are estimated to occur.^1^ Ovarian cancer is highly heterogeneous in nature and includes different histological subtypes with distinct clinicopathological and genetic features; thus, it is often classified into type I and II tumours.^2^ Among them, high‐grade serous ovarian carcinoma (HGSOC; a major Type II tumour) is the most prevalent and the most aggressive subtype, accounting for three quarters of all ovarian cancer cases.^3^, ^4^, ^5^, ^6^ For now, surgery and traditional chemotherapy remains the most common treatment, but unfortunately, HGSOC is often diagnosed in the advanced stage, and some patients develop chemoresistance. Therefore, there is a clear need to expand the therapeutic arsenal for HGSOC.

Immunotherapy of anti‐programmed cell death (PD)‐1 therapy has recently become a pillar of modern treatments against advanced stage malignancies. However, only limited objective responses were observed.^7^ Identifying potential therapeutic markers associated with treatment response or resistance would allow tailoring of appropriate immunotherapy for different patient subgroups. Nevertheless, little is known about the immune milieu of HGSOC and how to utilize this information to aid in the decision‐making of immunotherapy.

Utilizing consensus non‐negative matrix factorization (cNMF), we dissected the mRNA profile of HGSOC and identified an immune‐specific class with specific biological traits. This class exhibited markedly activated immune cells, enhanced cytolytic activity and enrichment of immunotherapy‐predictive gene signatures. We further refined this class into two robust microenvironment‐based subtypes characterized by either immunoactivation or carcinoma‐associated fibroblasts (CAFs). Our findings shed light on the immunogenomic landscape of HGSOC, offering insights to further personalized and precise treatments.

## 2 MATERIALS AND METHODS

### 2.1 Patients and samples

For the purpose of the study, we analysed the gene expression profiles from a total of 900 HGSOC human tumour samples, including a training cohort of 418 samples from The Cancer Genome Atlas (TCGA), profiled by RNA‐Seq, and two public data sets profiled by microarray that included 482 HGSOC samples for further validation (GSE9891: Tothill cohort^8^; GSE32062: Yoshihara cohort^9^). Methylation data were downloaded from UCSC Xena (https://xena.ucsc.edu/) which contains DNA methylation β values of 431 HGSOC samples with 417 tumour samples and 14 adjacent‐normal samples assessed by TCGA using the Illumina Infinium HumanMethylation27 platform (Illumina, San Diego, CA). Mutation data (mc3.v0.2.8.PUBLIC.maf.gz) were downloaded from PanCanAtlas and filtered for the HGSOC tumour type, which provided 411 samples; 275 samples were intersected with the mRNA profile and selected for downstream analysis. Copy number alteration (CNA) analysis results for HGSOC were downloaded from the FIREBROWSE (http://firebrowse.org) standard analyses procedure under the archive gdac.broadinstitute.org_OV‐TP.CopyNumber_Gistic2.Level_4.

### 2.2 Bioinformatics

We profiled the mRNA expression matrix from the raw, paired‐end reads in FASTQ format for 418 HGSOC samples first. Raw counts of mRNAs were transformed to fragments per kilobase of non‐overlapped exons per million mapped reads (FPKM) and low expressions were further removed to reduce noise. Tumour, stromal and immune cell transcriptome profiling data in the training TCGA set were microdissected virtually using unsupervised cNMF. Immune‐related gene signatures representing different immune statuses or immune cells were used to characterize immune‐specific subtypes by single‐sample gene set enrichment analysis (ssGSEA) and Nearest Template Prediction (NTP). Tumour Immune Dysfunction and Exclusion (TIDE), subclass mapping and pRRophetic algorithm were harnessed to predict the clinical response to immune checkpoint blockade and chemotherapeutic drugs. For detailed descriptions of data acquisition and methods, see the Supplementary Materials, available at *Cell Proliferation*.

### 2.3 Benchmarking of cell lines

#### 2.3.1 Cell culture

SKOV3 cells were obtained from the American Type Culture Collection (ATCC, Manassas, VA) and grown in DMEM supplemented with ampicillin (0.069 g/L)‐streptomycin (0.11 g/L) and 10% FBS. Cells were incubated in a humidified 5% (v/v) CO_2_ atmosphere at 37°C. For in vitro experiments, we treated SKOV3 cells with PTX at a concentration of 2.5 nM, 5 nM, and 10 nM.

#### 2.3.2 Cell transfection with siDIRAS3

DIRAS3 siRNA (Guangzhou RiboBio Co., Ltd., Guangzhou, P. R. China) was transfected using Exfect Transfection reagent (Guangzhou RiboBio Co., Ltd.) according to manufacturer's instructions.

#### 2.3.3 CCK8 assay

SKOV3 cells were cultured in 96‐well plates for 24 hours and incubated with siRNA and/or PTX for 24 hours. To evaluate cell survival, CCK‐8 solution was added to SKOV3 cells and incubated for 1 hour at 37°C. The absorbance at 450 nm was then determined using a microplate reader (iMark; Bio‐Rad Laboratories, Hercules, CA).

#### 2.3.4 Protein extraction and western blotting

Cell lysates were obtained by incubating cells in protein lysis buffer (125 mmol/L Tris and 2% SDS; pH 6.8). Proteins were then separated by 10% SDS‐PAGE and transferred to PVDF membranes (Millipore, Burlington, MA). The membranes were blocked with 2% bovine serum albumin in TBST (50 mmol/L Tris, 150 mmol/L NaCl, 0.5 mmol/L tris‐buffered saline, and Tween‐20; pH 7.5) and incubated with specific STAT1 (1:1,000; 10144‐2‐AP; Proteintech, Rosemont, IL) and p‐STAT1 (1:1,000; 7649; Cell Signaling Technology, Danvers, MA) antibodies at 4°C overnight. After washing, the blots were incubated with HPR‐conjugated secondary antibodies for 1 hour at 37°C. An enhanced chemiluminescence kit (36222ES60; Yeasen, Shanghai, PR China) was used to detect the immunoreactive proteins. Finally, the membranes were stained with Naphtol blue (030H0125; Sigma‐Aldrich, St. Louis, MO) as control, after which protein signals were semi‐quantified by analysing the signals of different groups using Image J (NIH, Bethesda, MD).

#### 2.3.5 RNA extraction and real‐time quantitative PCR (qPCR)

Total RNA from SKOV3 cells was isolated using TRIzol reagent, after which RNA was reverse transcribed to cDNA using a reverse transcription system kit (G490; abmGood, Vancouver, Canada). Then, qPCR was performed using a StepOnePlus Real‐Time PCR System (Applied Biosystems, Foster City, CA) with cDNA templates and SYBR Green qPCR Master Mix (B21202; Bimake, Houston, TX); the reaction mix consisted of 5 μL Master Mix, 0.3 μL forward primer, 0.3 μL reverse primer, 3.9 μL RNase‐free H_2_O, and 0.5 μL cDNA in a total volume of 10 μL. Primer sequences are listed in Supporting Information Table S1. qPCR was performed in triplicate, and each experiment was repeated at least three times.

### 2.4 Statistical analyses

All statistical tests were executed by R v3.5.2 with Fisher's exact test for categorical data, a two‐sample Mann‐Whitney *U* test for continuous data, one‐sided Fisher's exact test for over‐representation, and a log‐rank test Kaplan‐Meier curve and Cox regression for obtaining the hazard ratio (HR). Differences in immune‐estimated scores between the immune and non‐immune group were evaluated by a one‐tailed Mann‐Whitney U test. Differences in continuous data among multiple groups were evaluated by the Kruskal‐Wallis test. For all statistical analyses, a *P* < 0.05 was considered statistically significant.

## 3 RESULTS

### 3.1 Identification of a novel immune class in HGSOC

**FIGURE 1:**
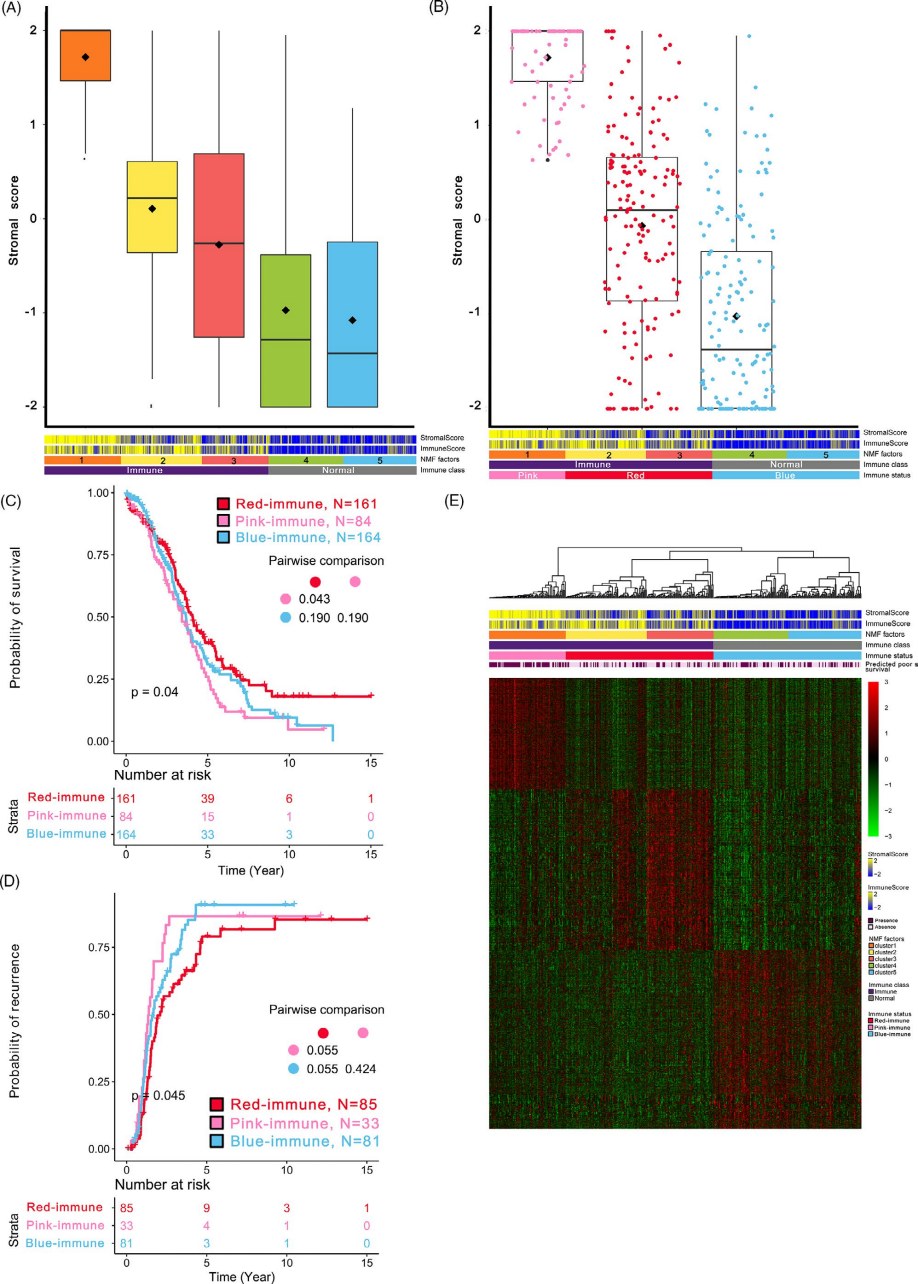
Quantification of the stromal enrichment levels of each cNMF factor and identification of the three immune‐specific subtypes of HGSOC. A, Boxplot of the stromal enrichment score of each cNMF factor. B, Boxplot of the stromal enrichment score of the three immune‐specific subtypes. C, Kaplan‐Meier curves of overall survival of the three immune‐specific subtypes. D, Kaplan‐Meier curves of recurrence time of the three immune‐specific subtypes. E, Heatmap of the gene expression profiles derived from unique significantly overexpressed genes based on pairwise comparison. *P* values was adjusted by Benjamini‐Hochberg in pairwise comparison of survival

We performed cNMF in the training cohort of 418 HGSOC samples from TCGA with factorization rank of 5 due to the Bayesian information criterion (BIC; Supporting Information Figure S1). Immune enrichment score (IES) was estimated for each sample in each cNMF factor (Supporting Information Figure S2A), and the factors were grouped as immune (252 samples) or non‐immune (166 samples) classes with a significantly higher IESs in immune class (*P* < .001; Supporting Information Figure S2B). Patients belonging to the immune class showed significant enrichment of immune cells signatures, *that is* T cells, B cells, cytotoxic cells, tertiary lymphoid structures (TLS), macrophages, as well as T.NK metagene and PD‐1 signalling signatures (Figure 1). NTP using a 51‐gene signature indicated that the immune group was highly associated with stem‐like samples (*P* < 0.001), which was consistent with a previous study.^10^ Differential expression analysis identified 250 genes as significantly dysregulated (DEGs) (Supplementary Supporting Information Figure S2C, Supporting Information Table S2). Among which, 61 immune‐related genes that were overexpressed compared with the non‐immune group were identified, including adaptive immune response genes, such as granzyme B (GZMB), CD8A, and CXCL11. Additionally, univariate Cox regression picked 21 survival‐related genes such as GZMB (HR = 0.86, 95% CI = [0.76, 0.98], *P* = 0.02), SH2D1A (HR = 0.67, 95% CI = [0.48, 0.94], *P* = 0.02) and CTLA4 (HR = 0.73, 95% CI = [0.53, 0.99], *P* = 0.04; Supporting Information Figure S3A). Gene ontology analysis indicated enrichment of immune‐related functions for the deregulated genes (all, FDR < 0.001; Supporting Information Figure S3B). However, no difference was observed in overall survival (OS) nor disease‐free survival (DFS) between two groups (Supporting Information Figure S3C, D). We theorized that there are other factors in the immune group that affect the prognosis of HGSOC patients.

### 3.2 The Immune group can be refined into two microenvironment‐based subtypes

The intricate interactions between cancer cells and the tumour microenvironment can drive host immune responses to produce growth factors that promote cancer progression and metastasis.^11^ Thus, we analysed the type of immune modulation occurring in response to the tumour microenvironment of HGSOC patients within the immune group. We estimated stromal enrichment score (SES) for each sample in each cNMF factor (Figure 1A) and refined the immune group into two subtypes termed pink‐ and red‐immune subtypes (Figure 1B, see more details in METHODS), while the non‐immune group was termed blue‐immune for lack of both IES and SES. Both IES and SES were significantly different among the three immune‐specific subtypes (all, *P* < 0.001). Moreover, we observed significant difference in OS and DFS among three subtypes (all, *P* < 0.05; Figure 1C, D). The red‐immune subtype showed more favourable prognosis compared with the other two subtypes, whereas the pink‐immune subtype showed the worst (red vs. pink: *P* = 0.014 for OS, *P* = 0.035 for DFS; red vs. blue: *P* = 0.036 for DFS). NTP using a 31‐gene signature also demonstrated a poor prognosis for the pink‐immune subtype (*P* < 0.001; Figure 1E).

**FIGURE 2:**
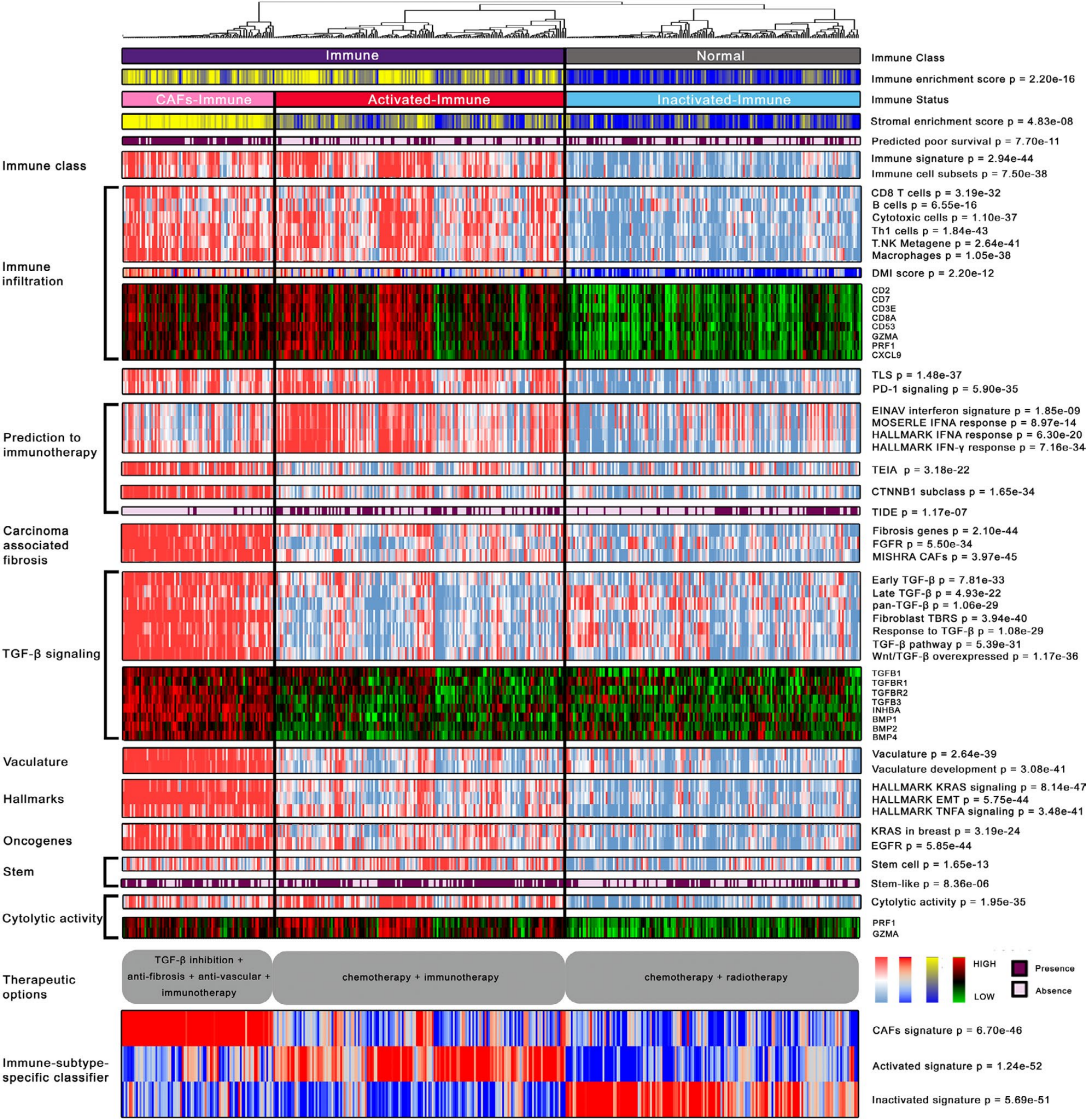
Characterization of the molecular landscape of the immune‐specific subtypes of HGSOC. A total of 418 HGSOC samples from TCGA training cohort were separated into immune (n = 252) and non‐immune groups (n = 166) by consensus non‐negative matrix factorization; the immune nature of the former was supported by gene signatures that reflect immune cells, infiltration and PD‐1 signalling. Immune group contains two microenvironment‐based subtypes, the activated‐immune (n = 166) and CAFs‐immune (n = 86) subtypes. The CAFs‐immune subtype was enriched for transforming growth factor beta 1 signalling, carcinoma‐associated fibroblasts and vasculature development that mediate immunosuppression. In the comprehensive heatmap, IES and SES were estimated by ‘estimator’ approach, continuous scores for molecular pathways were calculated by single‐sample gene set enrichment analysis, continuous expression level for genes was represented by log2‐transformed FPKM values, and binary classification of molecular phenotype was predicted by NTP algorithm; continuous values were further z‐scored and presented in the heatmap. High and low estimated IES and SES are presented in yellow and blue, respectively; high and low single‐sample enrichment scores as well as DNA methylation‐based immune infiltration scores are represented in red and blue, respectively; gene expression level was mapped to red and green colour range; the presence of feature predicted by NTP was indicated in purple. Opportunities for personalized therapy of each subtype are portrayed below in grey round box, and the single‐sample enrichment score of immune subtype‐specific classifier was drawn in the bottom of this heatmap. DMI, DNA methylation‐based immune infiltration; TLS, tertiary lymphoid structure; IFN: interferon; TIDE: tumour immune dysfunction and exclusion; TEIA: tumour escape from immune attack; CAFs: carcinoma‐associated fibroblasts; EMT: epithelial‐mesenchymal transition; TNFA: tumour necrosis factor‐alpha

Red‐immune subtype demonstrated significant enrichment of immune cell signals, immune‐related pathways, and DNA methylation‐based immune infiltration scores; IFN signatures that were associated with pembrolizumab response were also enriched in this subtype (Figure 2, Supporting Information Figure S4A). Although the pink‐immune subtype was associated with immune‐related pathways, it was also significantly characterized by immunosuppressive components, such as TGF‐β signalling, CAFs, and a frequent occurrence of epithelial‐mesenchymal transition (EMT) (Figure 2, Supporting Information Figure S4B). We used MCPcounter algorithm to compare tumour immune microenvironments (TIMEs) among three immune‐specific subtypes. Likewise, we found significant elevations in the proportion of cells involved in immune infiltration (eg T cells, CD8 T cells, Natural killer cells) in both red‐ and pink‐immune subtypes (all, *P* < .001), whereas the proportion of fibroblasts was significantly higher in pink‐immune subtype than that of in red‐immune subtype (Supporting Information Figure S4C).

Furthermore, red‐immune subtype was associated with the highest abundance of M1 macrophages (Supporting Information Figure S5A). Early studies in mouse tumour models indicated IFN‐α production would generate a long‐lasting antitumor response and IFN‐γ is a promising marker of response to immune checkpoint blockage in some solid tumours.^12^, ^13^ Consistently, most patients within red‐immune subtype were positively predicted to respond to immune checkpoint blockade by TIDE algorithm (*P* < 0.001); thus, we renamed this group ‘activated‐immune subtype’ (n = 166). Nevertheless, pink‐immune subtype was dominated by pro‐tumour M2‐polarized macrophages (Supporting Information Figure S5B) and a significantly higher M1 to M2 ratio compared with red‐immune subtype (Supporting Information Figure S5C). This subtype exhibited highly expressed CTNNB1 signalling (Figure 2), which suggests resistance to immunotherapies.^14^ TIDE algorithm demonstrated few samples within this subtype (11%, 10/86) can respond to immunotherapy and a 16‐gene signature of immune escape from immune attack was enriched in this group (all, *P* < 0.001, Figure 2); therefore, we designated this subtype as ‘CAFs‐immune’ (n = 86) and renamed the remaining blue‐immune subtype as ‘inactivated‐immune’ (n = 166).

Because immune checkpoint inhibitors have not yet been approved as a routine drug for HGSOC, we used subclass mapping, in addition to TIDE prediction, to compare the expression profiles of our defined immunophenotypes with another published data set containing 47 patients with melanomas that responded to immunotherapies.^15^ Interestingly, the immune class, especially the activated‐immune subtype, was more promising to anti‐PD‐1 therapy (Bonferroni‐corrected *P* < 0.05; Supporting Information Figure S6A,B, Supporting Information Table S3).

### 3.3 Validation of the immune‐specific subtypes using two independent cohorts

We identified 5,156 unique significantly overexpressed genes as immune subtype‐specific classifier by subtype pairwise comparison (Figure 1E, Supporting Information Table S4). The robustness of these immune‐specific subtypes was evaluated in two independent cohorts. First, we applied the 250 DEGs to the Tothill cohort containing HGSOC samples from the Australian ovarian cancer study and identified two groups with significantly different IESs (*P* < 0.001; Supporting Information Figure S7A,B). Similar to TCGA training cohort, 222 samples were supervised divided into three immune‐specific subtypes with 40 samples in activated‐immune, 97 samples in CAFs‐immune, and 85 samples in inactivated‐immune based on immune subtype‐specific classifier. These three subtypes also presented distinct IES and SES (all, *P* < 0.001; Supporting Information Figure S8A,B). Molecular characterization of these three immune‐specific subtypes confirmed significant enrichment of immune cell signatures in both activated and CAFs‐immune subtypes (Supporting Information Figure S8C); both subtypes also had highly enriched stem‐like samples (*P* < 0.001). The CAFs‐immune subtype showed enriched TGF‐β signalling, CAFs, and vasculature development (all, *P* < 0.001). TIDE showed that the activated‐immune subtype may be more sensitive to immunotherapy, while the CAFs‐immune subtype may fail (*P* < 0.001).

Next, we interrogated the Yoshihara cohort (n = 260) of Japanese population with serous ovarian cancer. Supervised analysis identified immune and non‐immune groups with distinct IESs (*P* < 0.001; Supporting Information Figure S9A,B), and three immune‐specific subtypes which comprised 54 samples in the activated‐immune, 98 samples in CAFs‐immune and 108 samples in inactivated‐immune subtype; a significantly different IES and SES were observed among these subtypes (all, *P* < 0.001; Supporting Information Figure S10A,B). Similar molecular enrichment and immunotherapeutic sensitivity (all, *P* < 0.001) to those derived from TCGA were observed (Supporting Information Figure S10C). We then analysed the predicted poor survival in both cohorts and again confirmed that CAFs‐immune subtype may be more fatal (both, *P* < 0.001). Therefore, our findings indicate that the molecular characteristics of the three immune‐specific subtypes were recapitulated in two independent data sets regardless of platform used (RNA‐Seq or microarray) or race.

### 3.4 Clinicopathologic characteristics of immune‐specific subtypes

We then explored the clinicopathologic characteristics of the three subtypes (Table 1). Consistent with the enrichment of vascular development pathways, most samples within CAFs‐immune subtype suffered from vascular invasion (*P* = 0.03). Macroscopic disease was observed in a few CAFs‐immune samples (*P* = 0.03). Univariate analyses were performed to assess the association between immune‐specific subtypes and OS for all patients and within each subtype of patients defined by the demographic or clinical factor sub‐categories (Table 2). The data suggested that activated‐immune subtype had a better OS than other two subtypes.

**TABLE 1:** Demographic and clinicopathologic characteristics of the HGSOC patients (TCGA; n = 411)

Clinicopathologic parameters	Frequency (%)	Immune‐specific subtypes			*P*
		Activated	CAFs	Inactivated	
Age (y)					.10
> 60	183 (45)	64	45	74	
≤ 60	228 (55)	99	39	90	
Ethnicity					.06
Hispanic or Latino	9 (2)	7	0	2	
Non‐Hispanic or Latino	237 (58)	93	55	89	
Missing	165 (40)	63	29	73	
Race					.66
White	362 (88)	143	76	143	
Asian	13 (3)	7	1	5	
Others	24 (6)	8	4	12	
Missing	12 (3)	5	3	4	
Neoplasm grade					.23
G1 + G2	48 (12)	15	14	19	
G3 + G4	355 (86)	144	69	142	
Missing	5 (2)	1	1	3	
Stage					.919
I + III	347 (84)	136	72	139	
IV	61 (15)	25	11	25	
Missing	3 (1)	2	1	0	
Lymphovascular invasion					.07
No	55 (13)	20	7	28	
Yes	104 (25)	48	22	34	
Missing	252 (62)	95	55	102	
Vascular invasion					.03^a^
No	47 (11)	19	4	24	
Yes	64 (16)	28	16	20	
Missing	300 (73)	116	64	120	
Residual disease					.03^a^
No macroscopic disease	73 (18)	27	7	39	
1‐20 mm	220 (54)	82	52	86	
>20 mm	74 (18)	30	19	25	
Missing	44 (10)	24	6	14	
Treatment outcome					.18
Complete remission/response	233 (57)	98	39	96	
Partial remission/response	46 (11)	14	15	17	
Progressive disease	31 (7)	15	7	9	
Stable disease	23 (6)	7	5	11	
Missing	78 (19)	29	18	31	
Disease‐free status					.75
Disease‐free	87 (21)	34	15	38	
Recurred/progressed	260 (63)	102	54	104	
Missing	64 (16)	27	15	22	
Primary tumour site					.14
Left	55 (13)	30	8	17	
Right	45 (11)	18	6	21	
Bilateral	286 (70)	107	62	117	
Missing	25 (6)	8	8	9	
Platinum status					.66
Too early	50 (12)	24	6	20	
Resistant	70 (17)	25	14	31	
Sensitive	164 (40)	66	31	67	
Missing	127 (31)	48	33	46	

^a^ Fisher's exact test *P* < 0.05.

**TABLE 2:** Characteristics of HGSOC patient survival based on immune‐specific subtypes

Immune class		Activated‐immune (A)			CAFs‐immune (C)			Inactivated‐immune (I)			Log‐rank test, Mantel‐Cox *P* (HR)		
	No. of subjects	N	Dead (%)	Median	N	Dead (%)	Median	N	Dead (%)	Median	A vs. C	A vs. I	C vs. I
Overall	409^a^	161	91 (57)	36.200	84	61 (73)	31.435	164	103 (63)	31.475	0.014^b^ (1.5)	0.15	0.19
Age (y)													
> 60	183	64	38 (59)	29.765	45	37 (82)	34.760	74	50 (68)	25.165	0.21	0.23	0.76
≤60	226	97	53 (55)	43.170	39	24 (62)	29.070	90	53 (59)	37.615	0.085 (1.53)	0.52	0.24
Neoplasm grade													
G1 + G2	48	15	8 (53)	40.370	14	10 (71)	22.500	19	14 (74)	56.500	0.046^b^ (2.61)	0.69	0.14
G3 + G4	359	145	82 (57)	35.990	70	50 (71)	34.360	144	86 (60)	27.415	0.083 (1.36)	0.17	0.55
Stage													
II + III	345	134	73 (54)	36.235	72	52 (72)	36.350	139	85 (61)	31.180	0.027^b^ (1.49)	0.088 (1.31)	0.43
IV	61	25	18 (72)	36.600	11	8 (73)	20.660	25	18 (72)	32.980	0.35	0.62	0.13
Lymphovascular invasion													
No	55	20	4 (20)	29.455	7	7 (100)	30.030	28	14 (50)	26.875	0.0027^c^ (5.75)	0.058 (2.84)	0.44
Yes	103	47	27 (57)	33.440	22	7 (32)	20.830	34	15 (44)	23.870	0.75	0.77	0.74
Vascular invasion													
No	47	19	7 (37)	38.040	4	3 (75)	37.060	24	11 (46)	21.745	0.69	0.2	0.48
Yes	64	28	13 (46)	38.685	16	5 (31)	22.340	20	8 (40)	29.865	0.9	0.75	0.87
Residual disease													
No macroscopic disease	73	27	10 (37)	41.590	7	4 (57)	29.010	39	16 (41)	33.940	0.49	0.91	0.63
1‐20 mm	219	81	53 (65)	36.200	52	39 (75)	39.405	86	60 (70)	29.125	0.22	0.12	0.82
>20 mm	74	30	21 (70)	28.615	19	16 (84)	19.450	25	17 (68)	28.350	0.42	0.7	0.12
Primary tumour site													
Left	54	29	13 (45)	41.950	8	6 (75)	37.695	17	12 (71)	39.060	0.06 (2.57)	0.29	0.28
Right	45	18	11 (61)	17.360	6	5 (83)	31.360	21	11 (52)	25.360	0.63	0.36	0.56
Bilateral	286	107	64 (60)	36.330	62	44 (71)	33.180	117	74 (63)	31.180	0.11	0.24	0.57
Platinum status													
Sensitive	163	65	31 (48)	49.640	31	20 (65)	44.970	67	44 (66)	43.890	0.26	0.077 (1.51)	0.69
Resistant	70	25	21 (84)	29.400	14	13 (93)	22.435	31	28 (90)	33.900	0.85	0.28	0.55

^a^ Two cases with NA (not available) clinical record.

^b^ < 0.05.

^c^ < 0.01, significant, based on log‐rank test *P* values.

### 3.5 Immune‐specific subtypes are associated with potentially targetable oncogenes or tumour suppressors

We investigated the relationship between the three subtypes and various well‐known oncogenic genes and tumour suppressors in ovarian cancer from cBioPortal and found EIF5A2 (*P* < 0.001), FGF1 (*P* < 0.001) and EGFR (*P* = 0.002) were highly expressed in the CAFs‐immune subtype. SPARC, a putative tumour suppressor whose high expression levels were found associated with advanced stage, low differentiation, lymph node metastasis and poor prognosis of ovarian cancer,^16^ was also significantly overexpressed in the CAFs‐immune subtype (*P* < 0.001). Downregulated tumour suppressors DIRAS3, DLEC1 and PEG3 (all, *P* < 0.001) were frequently observed in the immune group.

### 3.6 Immune group exhibits a low burden of chromosomal aberrations but significantly increased neoantigens, tumour mutation burden and microsatellite instability

Besides molecular features, the genomic landscape is also inextricably associated with anti‐tumour immunity; for example, TMB, MSI and neoantigen presence can trigger T‐cell responses,^17^, ^18^, ^19^ whereas aneuploidy may be correlated with immune evasion and reduced response to immunotherapy.^20^ The immune group showed a low burden of broad gains and losses (Supporting Information Tables S5 and S6). We observed significantly more neoantigens in the immune group than non‐immune group (*P* = 0.038), as well as TMB (*P* = 0.006). The immune group, especially the activated‐immune subtype, presented with a significantly higher MSI predictor score than that of the non‐immune group or other subtypes (all, *P* < 0.001). Additionally, the local immune cytolytic activity shows strong correlation with cytotoxic T cells and IFN‐stimulated chemokines that attract T cells.^17^ Interestingly, we observed a strong enrichment of the cytolytic activity score in immune group (*P* < 0.001).

### 3.7 Association between mutation signatures and immune group

We identified four significantly mutated genes (SMGs) for the immune group, including NF1, TOP2A and CDK12, and TOP2A has not been reported previously, and RB1 was the SMGs for non‐immune group (all, *q* < 0.05; Supporting Information Figure S11A). Tumours that show high correlation with contributor signature.3 tended to represent the immune group (*P* = 0.038; Supporting Information Figure S11B–E). Regardless of OS or DFS, tumours characterized by signature.3 showed better prognosis (OS, *P* = 0.0012; DFS, *P* = 0.0022; Supporting Information Figure S12A,B).

### 3.8 Recognition of the immune group by epigenetically regulated genes

In view of the general upregulation of immune‐related genes in the immune group, we further investigated whether such immunologic dysregulation could mirror epigenetic alterations. Using methylation 27k data, a total of 13,625 probes (9,987 genes) were mapped to promoter CpG islands. We identified 288 (18 immune‐related) epigenetically silenced genes and 375 (13 immune‐related) epigenetically activated genes (Supporting Information Tables S7 and S8). Separate supervised clustering of these genes could well distinguish the immune group from the non‐immune counterpart (all, *P* < 0.001; Supporting Information Figure S13A–C). Additionally, we jointly analysed differentially methylated probes/genes and DEGs, and 306 genes were explored to be simultaneously differentially expressed and methylated (Supporting Information Figure S14, Supporting Information Table S9). Supervised clustering of these 306 genes also isolated the immune and non‐immune groups (*P* < 0.001; Supporting Information Figure S13D), suggesting that epigenetic alteration play a critical role in the immunophenotype of HGSOC.

### 3.9 Differential chemotherapeutic responses among immune‐specific subtypes

**FIGURE 3:**
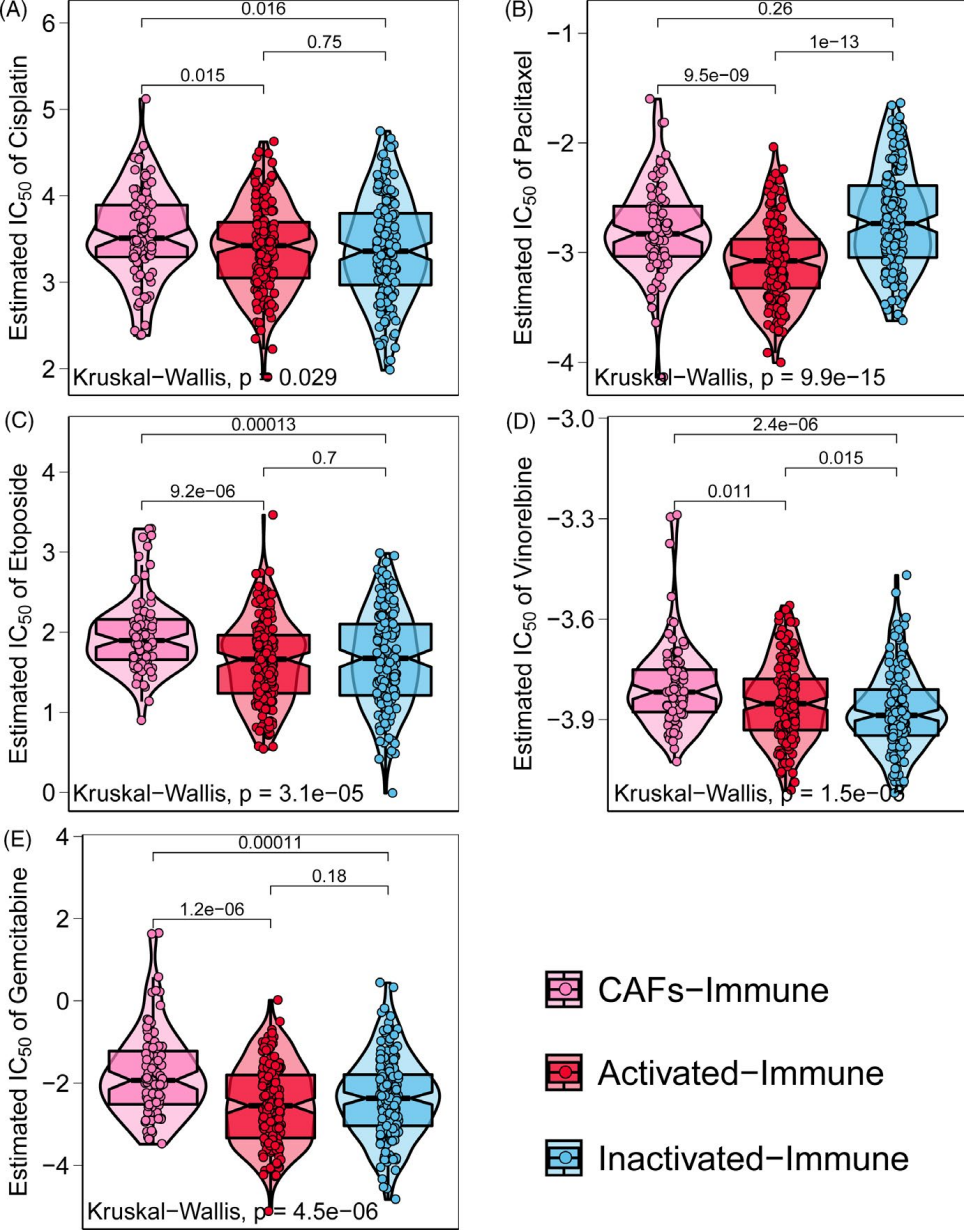
Distribution of estimated IC_50_ values of five chemotherapy drugs among the three immune‐specific subtypes. CAFs‐immune subtype was predicted to be resistant to all five drugs, while the activated‐immune subtype was sensitive to at least three drugs. Differences in estimated IC_50_ among three immune‐specific subtypes were evaluated by the Kruskal‐Wallis test. Pairwise comparisons were assessed by two‐sample Mann‐Whitney *U* test

To assess the response of the three immune‐specific subtypes to traditional chemotherapy, we estimated the IC_50_ values of each sample in TCGA data set for five chemotherapy drugs (ie cisplatin, PTX, etoposide, vinorelbine and gemcitabine), and significant response differences were observed (all, *P* < 0.05; Figure 3A‐E). Notably, the CAFs‐immune subtype was predicted to be resistant to all five chemotherapy drugs, whereas activated‐immune system was sensitive to at least three drugs. This finding is consistent with a recent study that reported how fibroblasts can block chemotherapy and immune cells can help reverse chemoresistance in ovarian cancer.^21^

### 3.10 Evaluation of the human ovarian cancer cell line SKOV3

**FIGURE 4:**
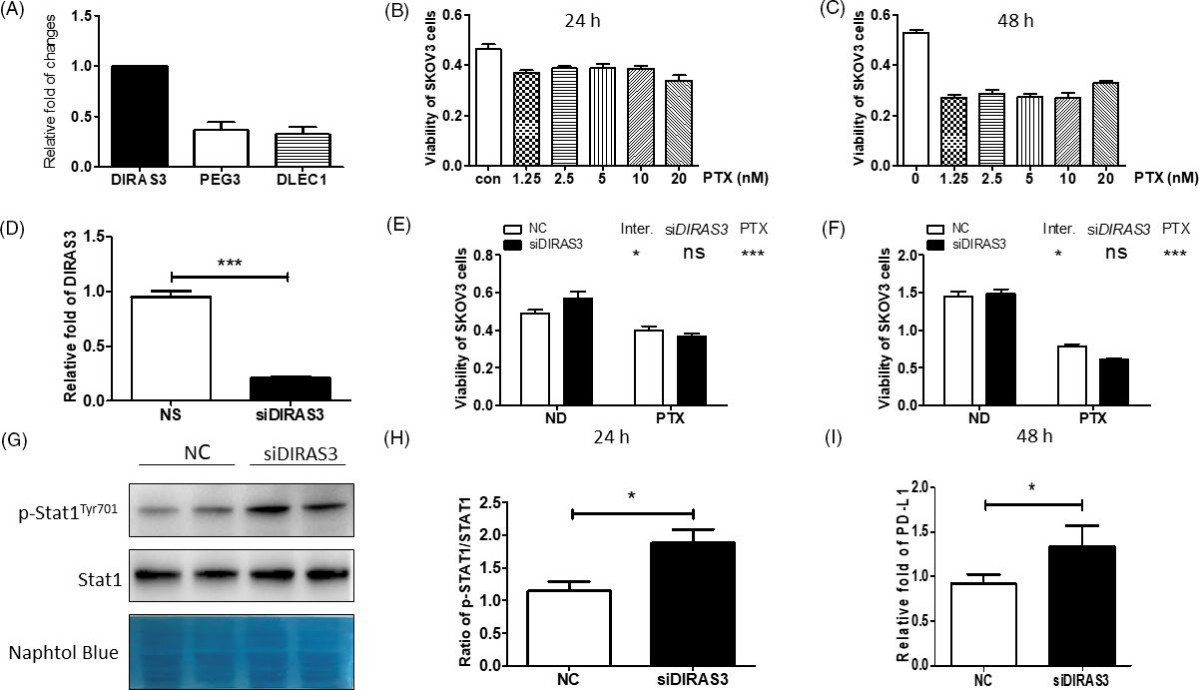
Benchmarking of cell lines, including characteristics of the human ovarian cancer cell line SKOV3, activation of STAT1 in DIRAS3‐knockdown SKOV3 cells, and increased PD‐L1 expression in DIRAS3‐knockdown SKOV3 cells. A, DIRAS3, PEG3 and DLEC1 expression in SKOV3 cells. B and C Survival of SKOV3 cells treated with PTX at different concentrations for 24 and 48 h. D, Efficiency of DIARS3 knockdown with siRNA transfection. E) and F) Survival of SKOV3 cells treated with 20 nM PTX after transfection with NC or siDIRAS3. *P < 0.05; ***P < 0.001 (two‐way ANOVA followed by a Bonferroni post hoc test). G, Protein levels of p‐STAT1 and STAT1 in SKOV3 cells transfected with NC or siDIRAS3. H, p‐STAT1/STAT1 ratio. *P < 0.05 (Student's *t* test). I, SKOV3 cells were transfected with NC or siDIRAS3 and PD‐L1 was quantified by real‐time PCR. *P < 0.05 (Student's *t* test)

We analysed the expression of frequently downregulated tumour suppressor genes in immune subtype (ie DIRAS3, PEG3, and DLEC1) in the human ovarian cancer cell line SKOV3 and found DIRAS3 was most highly expressed (Figure 4A). Therefore, DIRAS3 was chosen for further verification. PTX, which the activated‐ and inactivated‐immune subtypes were predicted to be sensitive to, whereas resistant to CAFs‐immune subtype, was used to treat SKOV3 cells at 1.25 mM to 20 mM for 24 and 48 hours, respectively, and no difference in cell viability at different PTX concentrations at either 24 or 48 hours (Figure 4B, C). However, 24 and 48 hours treatment of PTX induced cell death at a rate of 20% and 50%, respectively, which suggested SKOV3 cells were susceptible to PTX, indicating this cell line has similar features to the activated‐ and inactivated‐immune subtypes. We then knocked down DIRAS3 by using small interference RNA (siRNA; Figure 4D). Cell viability of siDIRAS3 or/and PTX‐treated SKOV3 cells was evaluated at different time points, and we found knockdown of DIRAS3 promoted cell death in PTX‐treated SKOV3 cells compared with negative control (NC) or PTX alone‐treated cells (Figure 4E, F).

### 3.11 Activation of IFN‐γ signalling in DIRAS3‐knockdown SKOV3 cells

To further evaluate whether the characteristics of DIRAS3‐knockdown SKOV3 cells are similar to the activated‐immune subtype, the activity of STAT1 was analysed in SKOV3 cells treated with siDIRAS3, which mimics the activated‐immune subtype in vitro. STAT1 was proven to be a direct target of IFN‐γ,^22^ and we found enhanced STAT1 phosphorylation (p‐STAT1) and increased p‐STAT1/STAT1 ratio in siDIRAS3‐transfected SKOV3 cells (SKOV3‐siDIRAS3; Figure 4G, H), indicating activated IFN‐γ signalling, which supported that SKOV3‐siDIRAS3 cells exhibited the characteristics of activated‐immune subtype.

### 3.12 Upregulation of PD‐L1 expression in DIRAS3‐knockdown SKOV3 cells

We then evaluated the levels of PD‐L1 in DIRAS3‐knockdown SKOV3 cells and found that there was an approximate 40% increase in PD‐L1 levels in SKOV3‐siDIRAS3 cells compared with NC‐treated cells (Figure 4I), indicating that DIRAS3‐knockdown SKOV3 cells are more immunoreactive and may be more sensitive to an anti‐PD‐L1 approach; such verification reinforced our prediction that the activated‐immune subtype of ovarian cancer was more sensitive to immunotherapy.

## 4 DISCUSSION

Although there have been reports of substantial response and increased survival after being treated with immune checkpoint inhibitors, FDA has not approved anti‐PD‐L1 immunotherapy specifically for HGSOC. Despite considerable efforts on ovarian cancer regarding these therapies, limited evidence of clinical utility has been reported.^12^ One study reported only one out of 17 ovarian cancer patients treated with anti‐PD‐L1 antibody BMS‐936559 achieved an objective response.^23^ In a single phase II trial that focused on ovarian cancer patients reported an overall response rate of 15% and a median progression‐free survival of 3.5 following treatment with anti‐PD‐1 antibody nivolumab.^24^ A similar response was achieved in a preliminary report of pembrolizumab administration in a phase Ib study to patients with PD‐L1‐positive ovarian cancer.^25^ The response rate in these trials is far from impressive, which drives the unmet need for identifying ideal candidates for ovarian cancer, and of course, HGSOC immunotherapy.

Our study identified a novel immune group of HGSOC which highly enriched samples with molecular characteristics that highly resemble those of cancers most responsive to immunotherapy, including high infiltration of immune cells and enrichment of PD‐1 signalling. However, inferring the response to immunotherapy solely by identifying the immune phenotype is unreliable. The intricate and dynamic interactions between tumour cells, immune cells, and other immunomodulators embedded in the microenvironment may either strengthen or weaken the immune response, thereby affecting the effectiveness of checkpoint inhibitors. Therefore, we incorporated the tumour microenvironment to further dissect the immune group, and obtained two microenvironment‐based subsets. Both activated‐immune and CAFs‐immune subtypes exhibited high expression of immune molecules; however, the former one exhibited antitumor immune features, such as enrichment of IFN signatures, active immune response genes and better prognosis, whereas the other was characterized by tumour‐promoting signals (eg activated stroma, anti‐inflammatory M2 macrophages). In particular, WNT/TGF‐β signalling pathway was activated in CAFs‐immune subtype; TGF‐β regulates tumour‐stroma interactions, EMT, angiogenesis and metastasis and can suppress the host anti‐tumour immune response, leading to a poor prognosis. Such divergence was also reflected in the prediction of clinical response to immune checkpoint blockade, where the activated‐immune subtype was more likely than the CAFs‐immune subtype to respond to immunotherapy.

Our findings have potential therapeutic implications for the rational design of combination therapy. For CAFs‐immune subtype, a combination therapy including TGF‐β inhibition as well as anti‐fibrosis, anti‐vascular and immunotherapy could be beneficial. This strategy is promising as a combination of TGF‐β inhibition and immunotherapy has been shown to induce complete responses in mouse models,^26^ and a phase 1b/2 clinical trial is currently underway to test the combination of a novel TGF‐β inhibitor, galunisertib, with nivolumab in treating advanced solid tumours (NCT02423343). Moreover, anti‐vascular therapies hold great promise for targeting the tumour microenvironment.^27^ For activated‐immune subtype, combining chemotherapy with immunotherapy may be the most effective treatment,^21^ while traditional chemotherapy and radiotherapy may be the current choice for the non‐immune group (inactivated‐immune subtype) because we failed to observe any significant enrichment favouring potential targeted therapies, including specific oncogenes or tumour suppressors. Furthermore, as we have also predicted the enrichment of a stem cell‐like phenotype in the immune group, perhaps this group would benefit from a promising strategy that targets cancer stem cells.^28^

Robustness of the immune‐specific subtypes was supported by their successful replication in two independent cohorts. To further support this, we analysed the characteristics of the ovarian cancer cell line SKOV3. In vitro experiments with DIRAS3‐knockdown SKOV3 allowed us to almost reproduce the activated‐immune subtype, because further investigation demonstrated that the activity of STAT1 was significantly induced in DIRAS3‐knockdown SKOV3 cells. Consistent with STAT1 levels, the expression of PD‐L1 was significantly induced in DIRAS3‐knockdown SKOV3 cells. Therefore, in vitro verification confirmed the activated‐immune subtype is more sensitive to chemotherapy and immunotherapy.

In conclusion, we introduced a novel immune group in HGSOC that contains two robust microenvironment‐based subtypes with distinct likelihoods of response to immunotherapies and who might represent ideal immunotherapy candidates. These findings warrant further investigations in larger HGSOC cohorts receiving immune checkpoint therapies.

## CONFLICT OF INTEREST

The authors have no conflict of interest.

## AUTHOR CONTRIBUTIONS

Conceptualization, XL and FY; methodology, XL and LJ; software, X.L and YZ.; validation, XL, CJ and JY; formal analysis, XL, CJ, and LJ; investigation, XL, CJ, LJ Y‐JZ, JM, and JG; resources, TL, JY, and FY; data curation, X.L and CJ.; writing—original draft preparation, XL, C.J, and Y‐JZ; writing—review and editing, JG and FY; visualization, XL and YZ; supervision, TL, JY, and FY; project administration, TL, JY, and FY; funding acquisition, FY All authors have read and agreed to the published version of the manuscript.

## ETHICAL STATEMENT

As the data (TCGA and GEO data sets) are publicly available, no ethical approval is required.

## Supporting information

Figure 1

Figure 2

Figure 3

Figure 4

Figure 5

Figure 6

Figure 7

Figure 8

Figure 9

Figure 10

Figure 11

Figure 12

Figure 13

Figure 14

Tables S1–S9

## Data Availability

Raw data for this study were generated at TCGA with cancer type of OV, and GEO database with Series ID of GSE9891, and GSE32062. Derived data supporting the findings are available from the corresponding authors [FY] on reasonable request.
