# Supplementary figures and images for: Tumour microenvironment‐based molecular profiling reveals ideal candidates for high‐grade serous ovarian cancer immunotherapy
Source: Cell Prolif. 2021 Jan 31;54(3):e12979. doi: 10.1111/cpr.12979 (PMC7941229; doi:10.1111/cpr.12979)

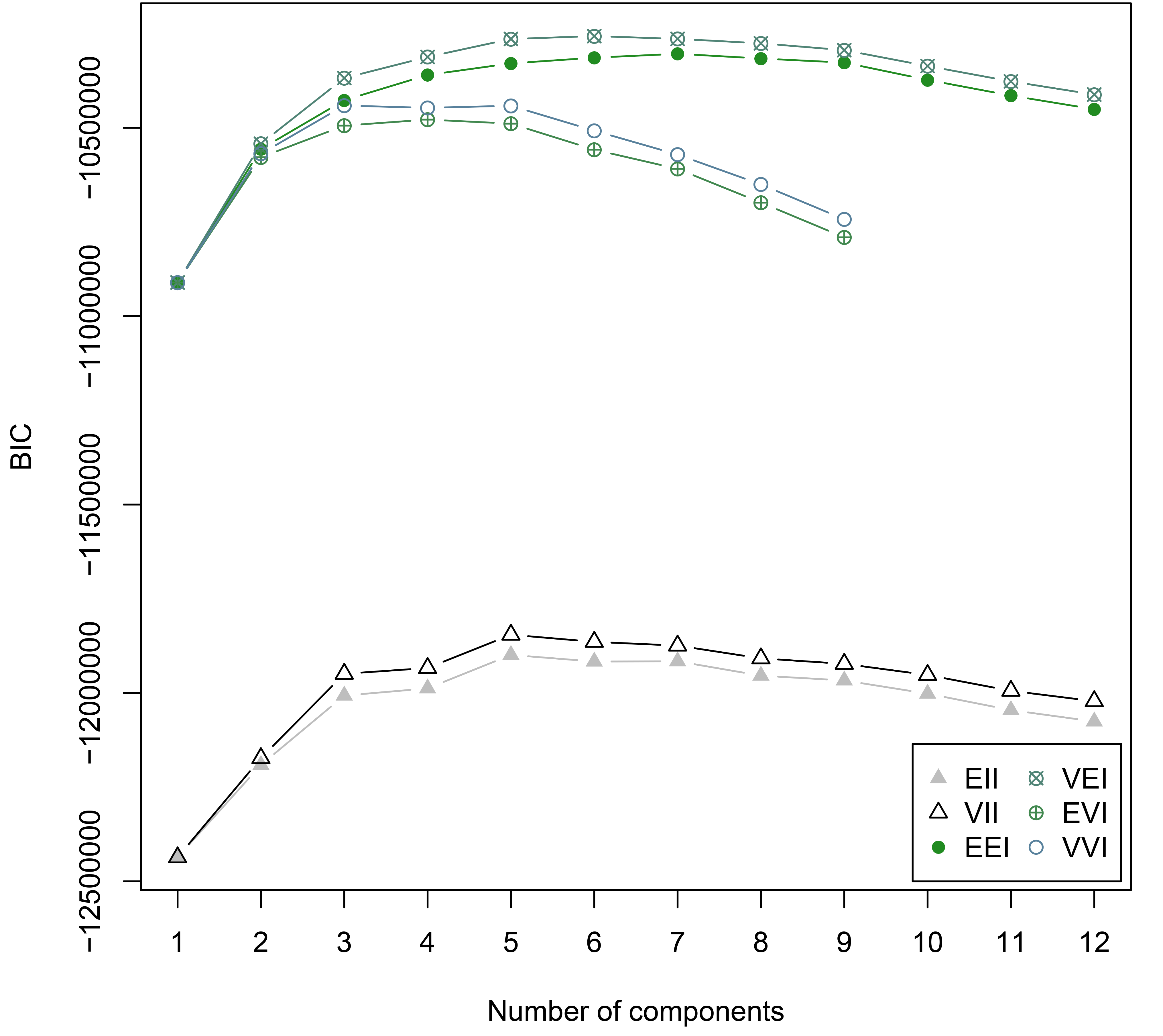

Supplement: Supplementary file 1 — Figure 1 [file CPR-54-e12979-s008.tif]

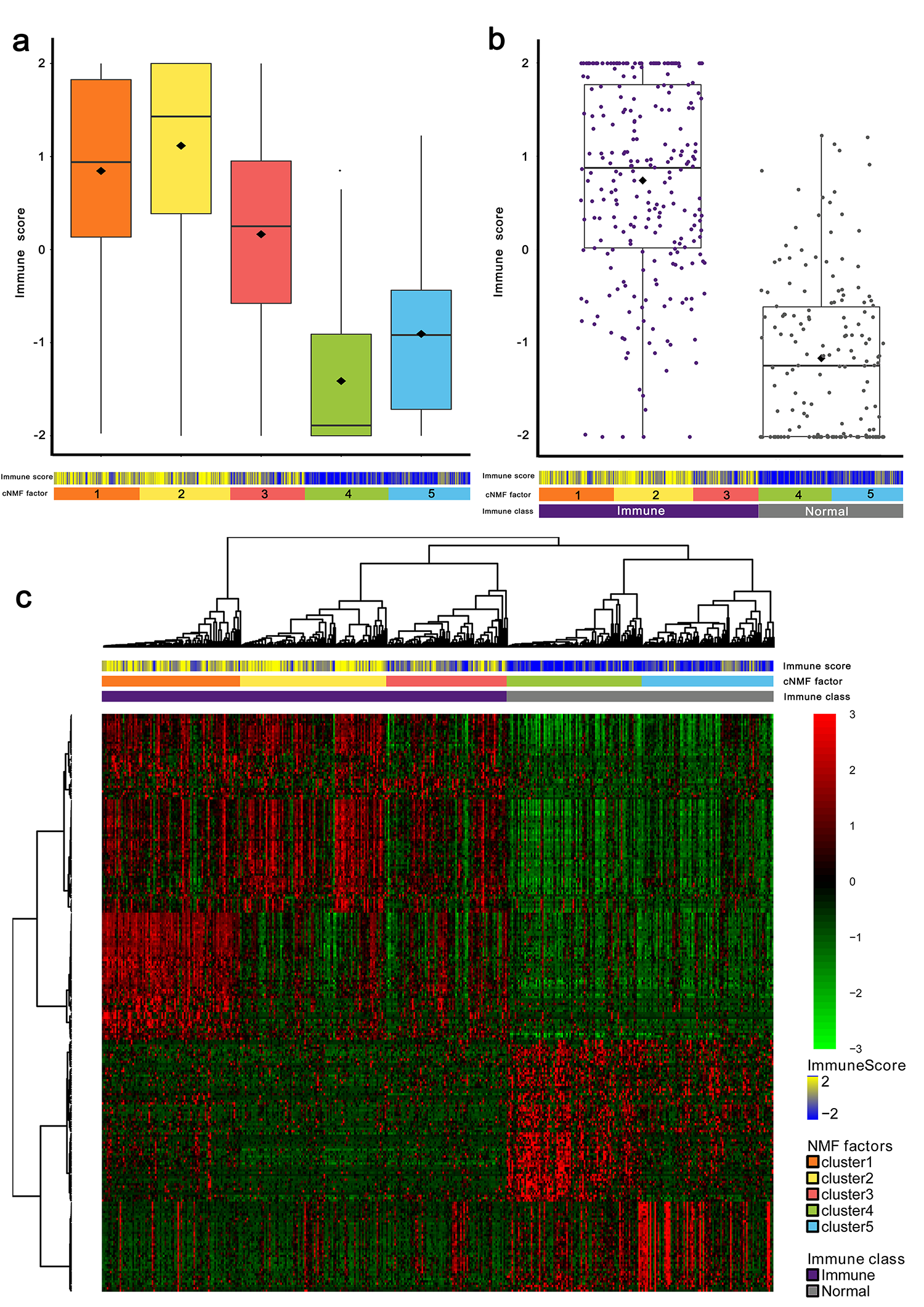

Supplement: Supplementary file 2 — Figure 2 [file CPR-54-e12979-s005.tif]

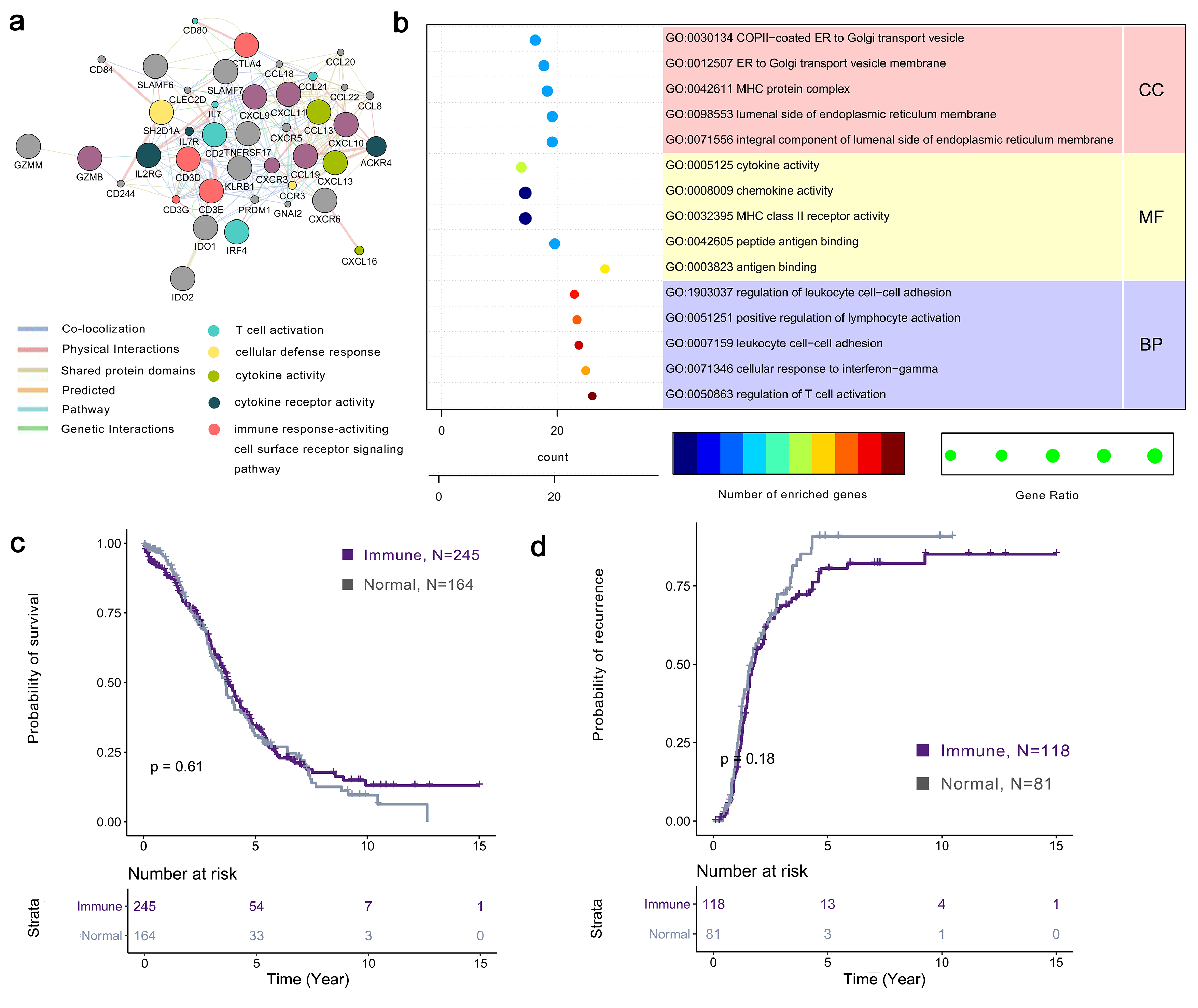

Supplement: Supplementary file 3 — Figure 3 [file CPR-54-e12979-s009.tif]

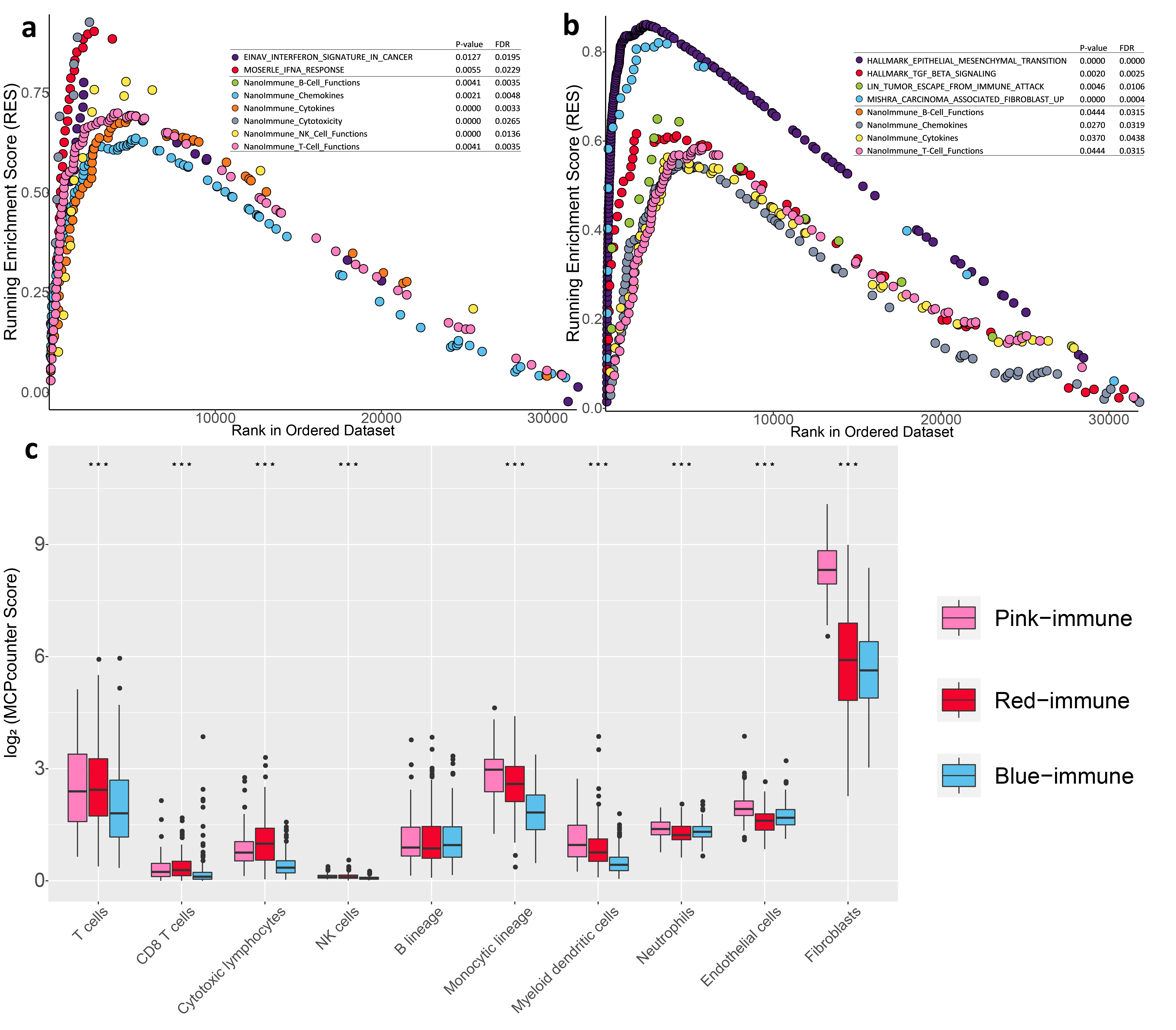

Supplement: Supplementary file 4 — Figure 4 [file CPR-54-e12979-s003.tif]

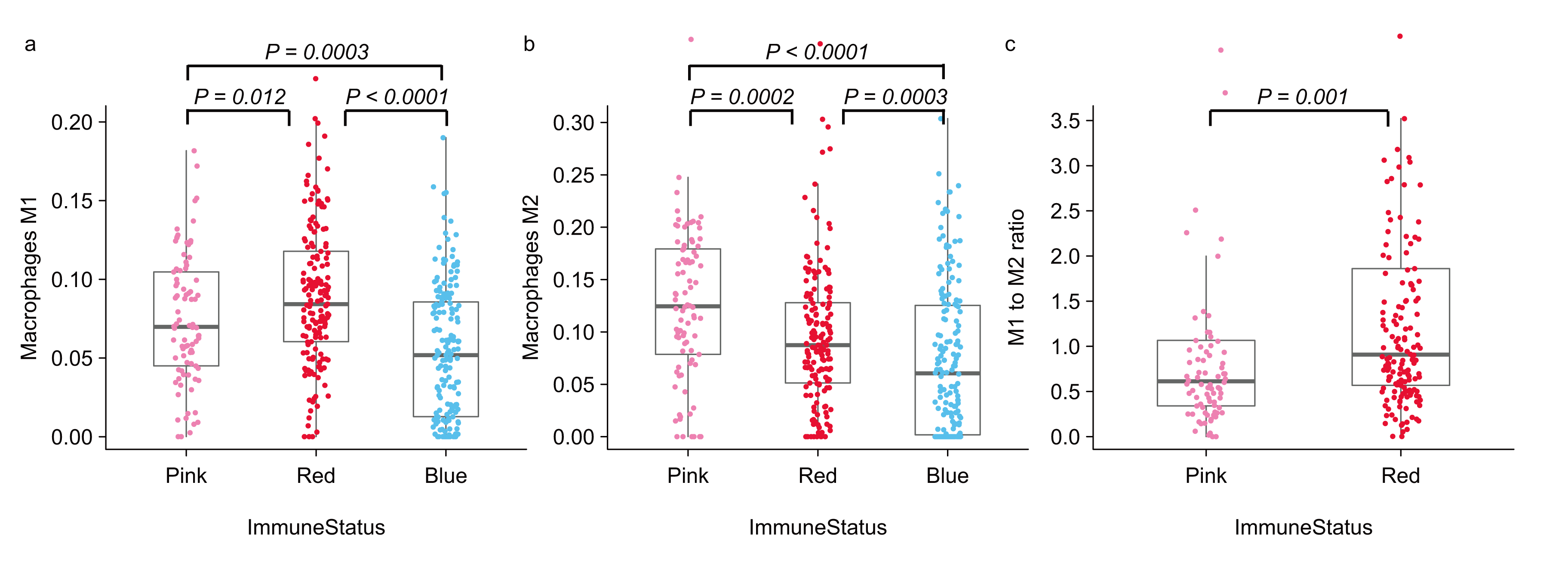

Supplement: Supplementary file 5 — Figure 5 [file CPR-54-e12979-s011.tif]

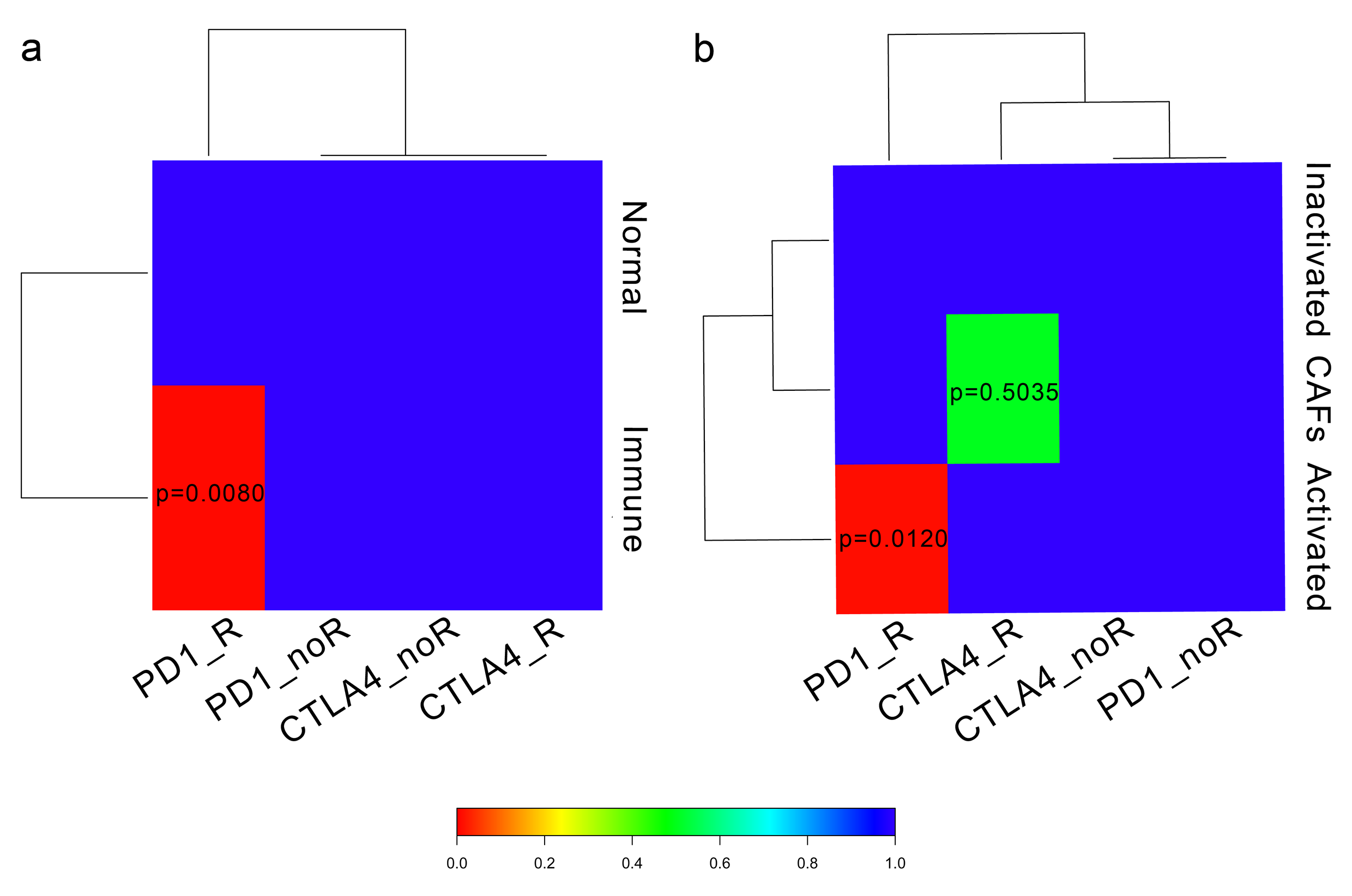

Supplement: Supplementary file 6 — Figure 6 [file CPR-54-e12979-s001.tif]

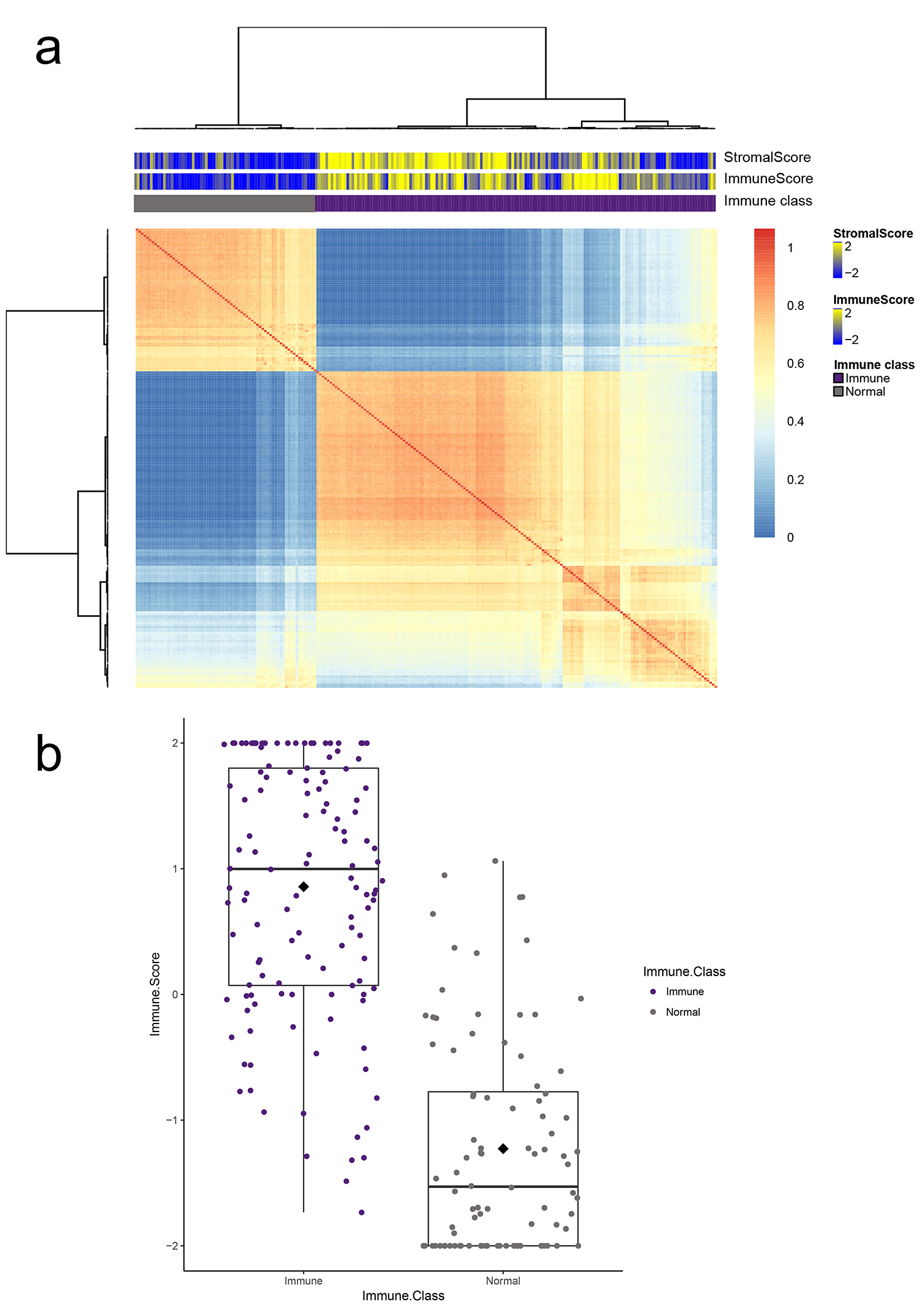

Supplement: Supplementary file 7 — Figure 7 [file CPR-54-e12979-s015.tif]

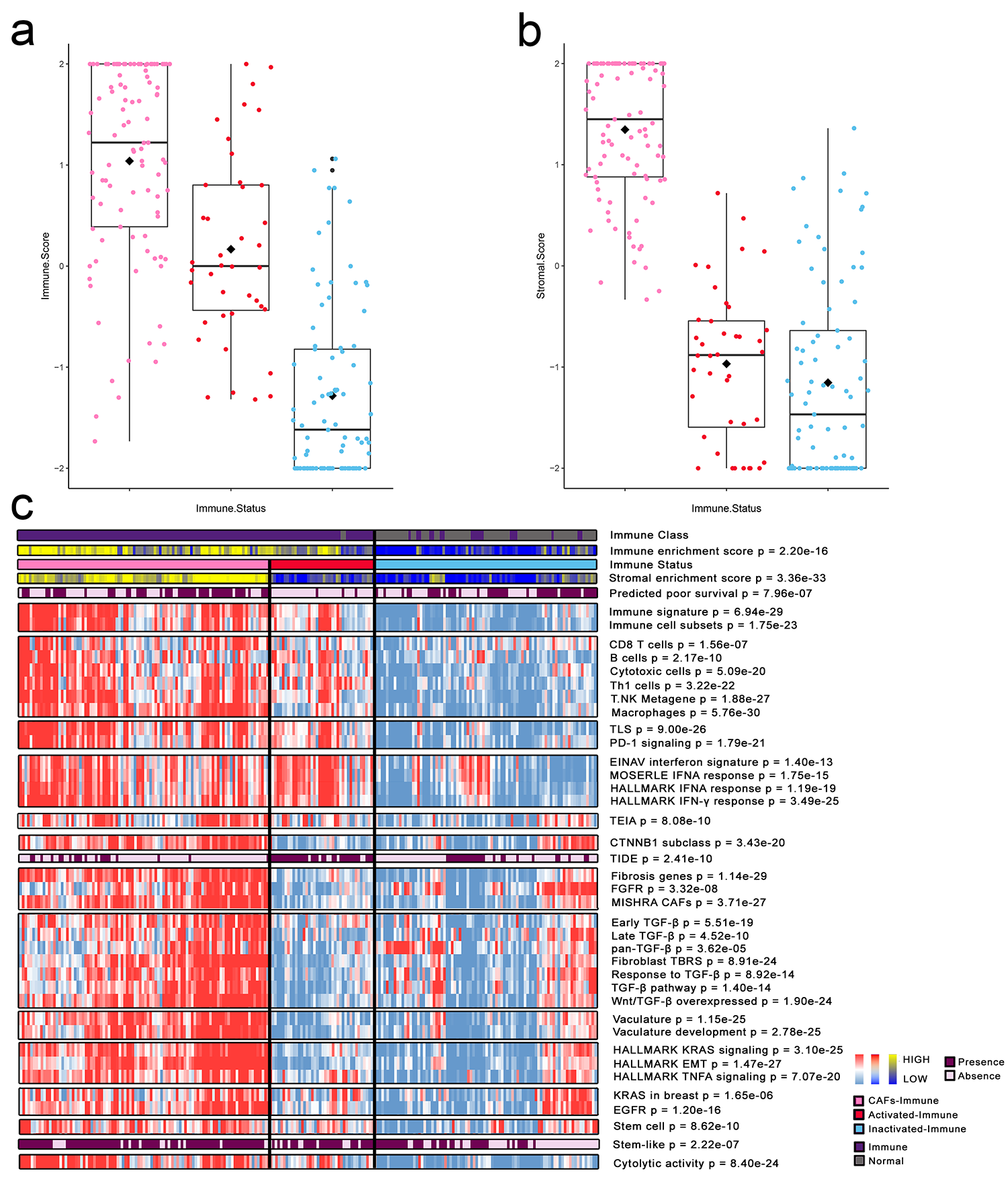

Supplement: Supplementary file 8 — Figure 8 [file CPR-54-e12979-s012.tif]

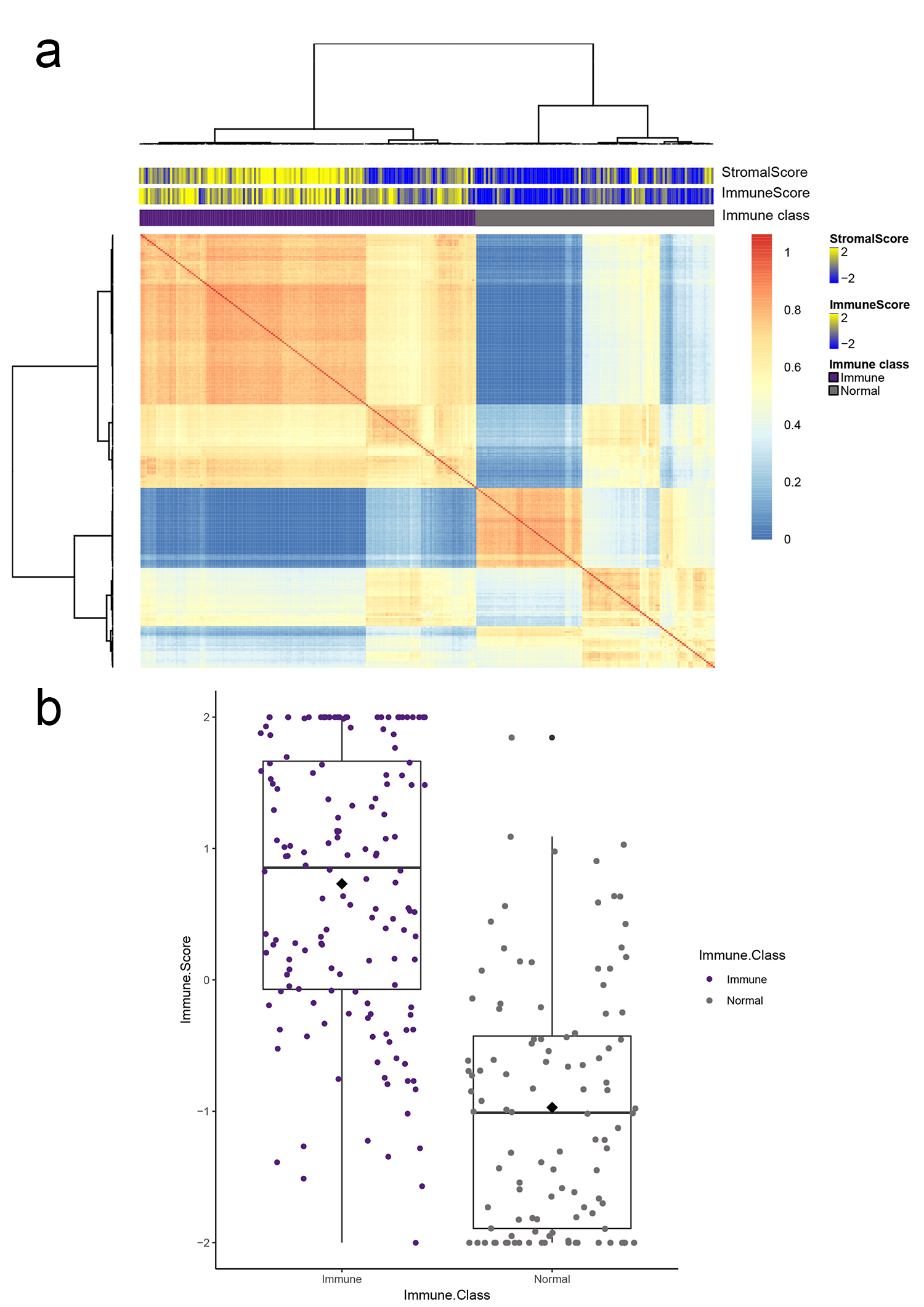

Supplement: Supplementary file 9 — Figure 9 [file CPR-54-e12979-s002.tif]

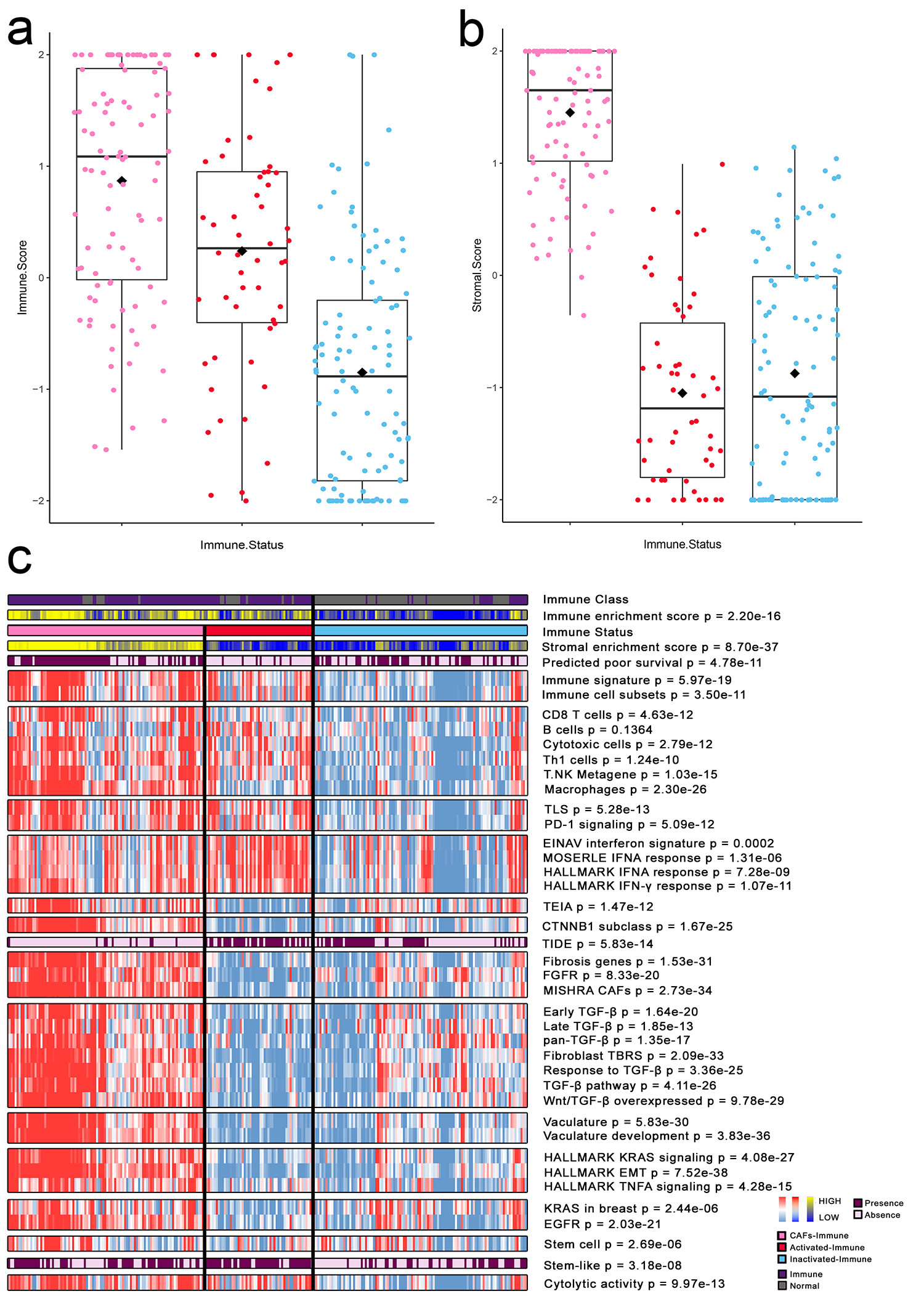

Supplement: Supplementary file 10 — Figure 10 [file CPR-54-e12979-s010.tif]

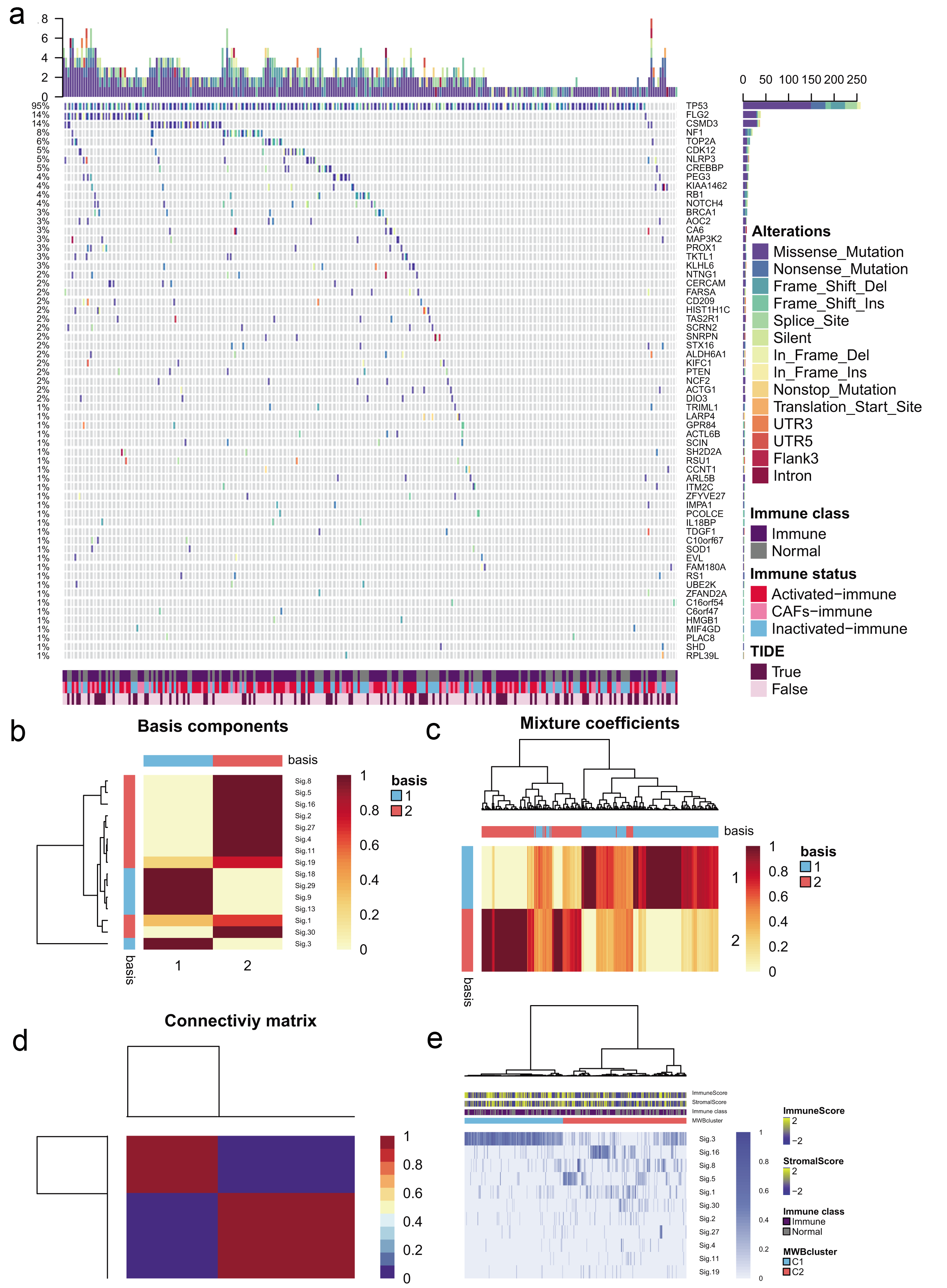

Supplement: Supplementary file 11 — Figure 11 [file CPR-54-e12979-s007.tif]

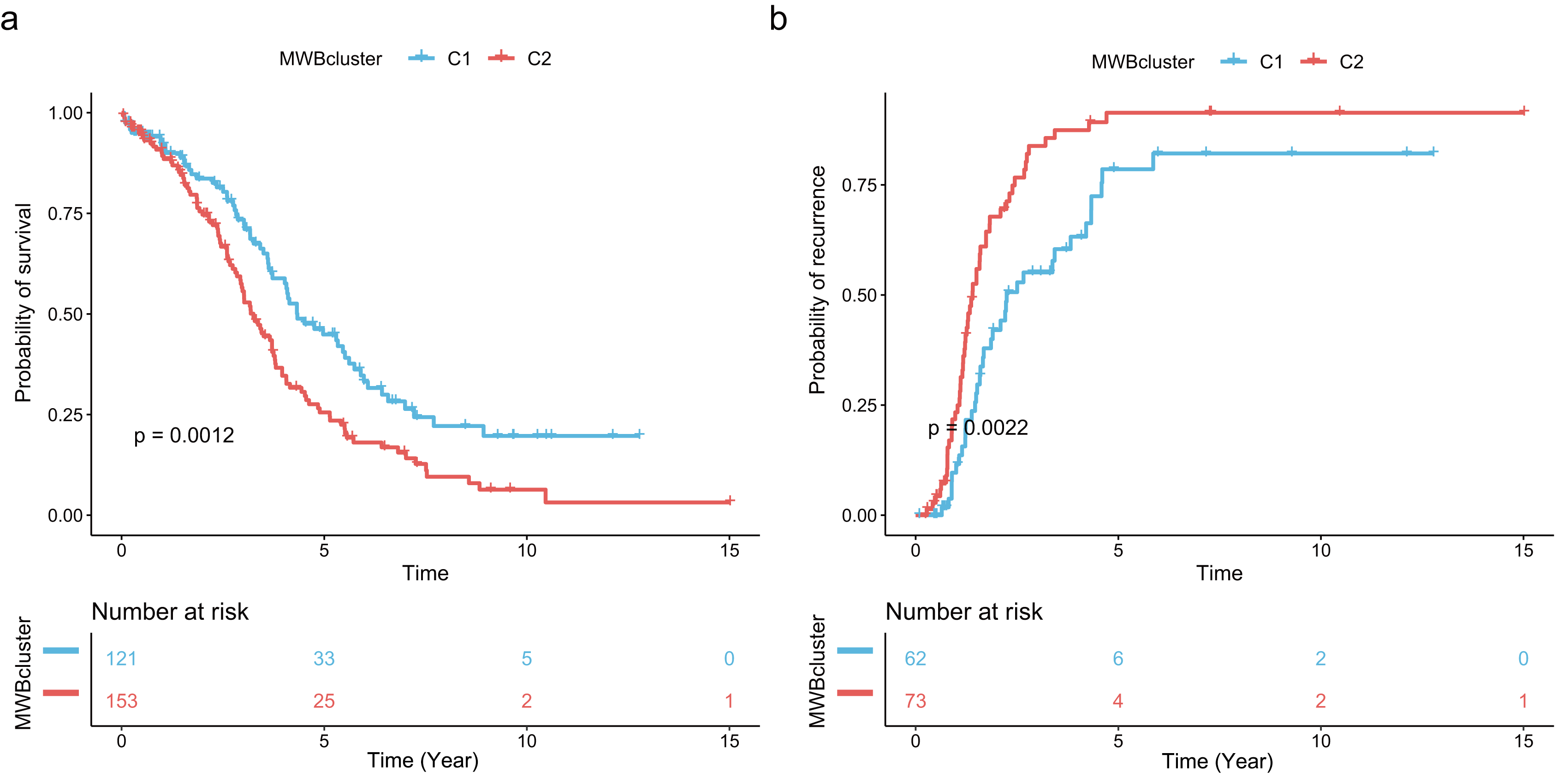

Supplement: Supplementary file 12 — Figure 12 [file CPR-54-e12979-s006.tif]

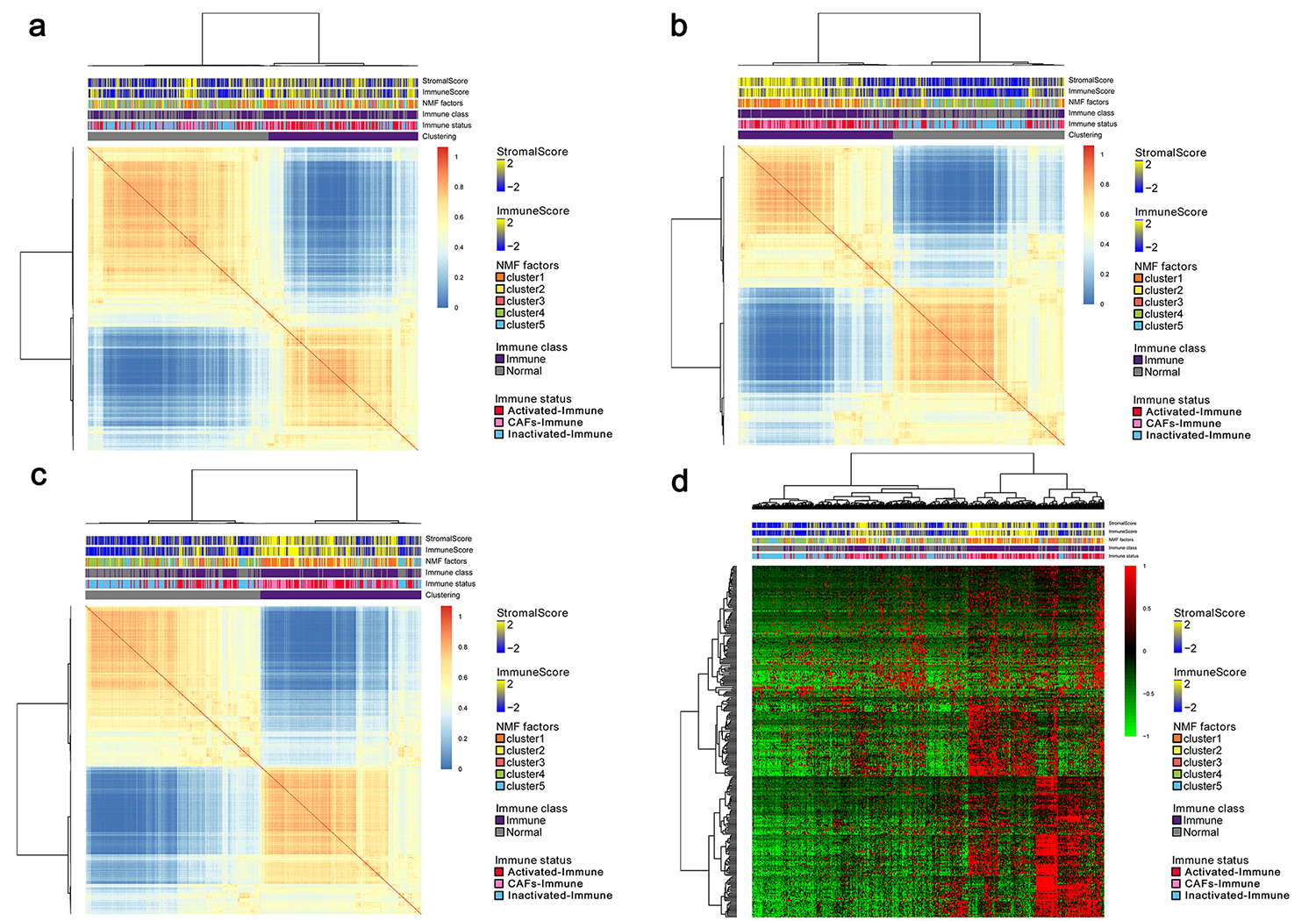

Supplement: Supplementary file 13 — Figure 13 [file CPR-54-e12979-s013.tif]

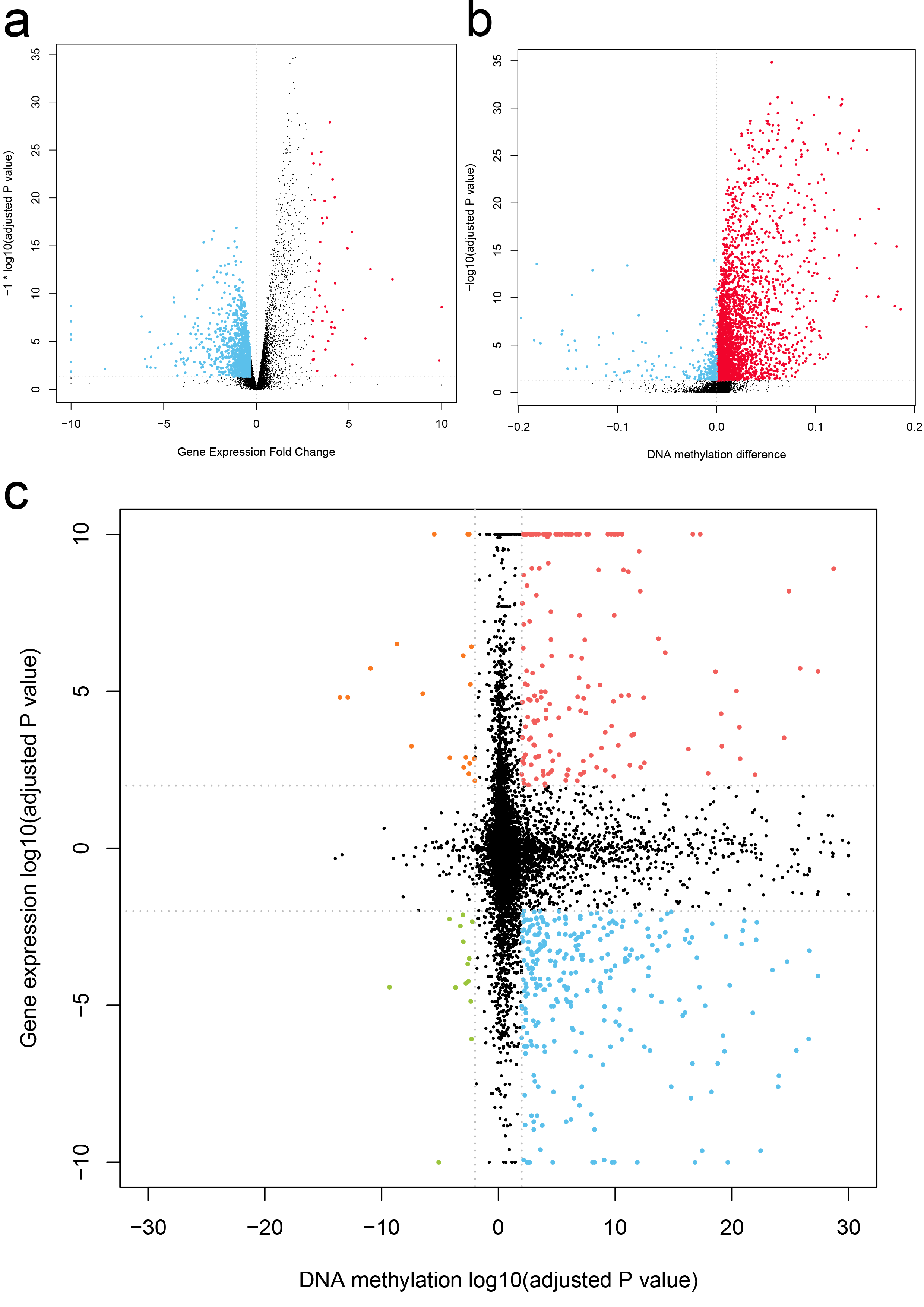

Supplement: Supplementary file 14 — Figure 14 [file CPR-54-e12979-s004.tif]
